# Bucephalidae (Digenea) from epinephelines (Serranidae: Perciformes) from the waters off New Caledonia, including *Neidhartia lochepintade* n. sp.

**DOI:** 10.1051/parasite/2013055

**Published:** 2013-12-19

**Authors:** Rodney A. Bray, Jean-Lou Justine

**Affiliations:** 1 Department of Zoology, Natural History Museum Cromwell Road London SW7 5BD UK; 2 UMR 7138, Systématique, Adaptation, Évolution, Muséum National d’Histoire Naturelle, Case postale 51 55 rue Buffon 75231 Paris Cedex 05 France

**Keywords:** Bucephalidae, *Neidhartia*, *Prosorhynchus*, *Epinephelus*, *Cephalopholis*, *Variola*, New Caledonia

## Abstract

Many bucephalid species, mainly of the subfamily Prosorhynchinae, have been described from epinepheline serranids (groupers) throughout the World’s Oceans. In this paper eight named prosorhynchine species are described and/or illustrated from epinepheline fishes from New Caledonia. *Neidhartia lochepintade* n. sp. in *Epinephelus chlorostigma* differs from other *Neidhartia* spp. in various combinations of distinct body-size, rhynchus size, previtelline and pre-mouth distance, post-testicular distance, cirrus-sac reach and egg-size. Other species are: *Neidhartia haywardi* Bott, Miller & Cribb, 2013 in *Plectropomus leopardus*; *Neidhartia tyleri* Bott, Miller & Cribb, 2013 in *Plectropomus leopardus* and *Plectropomus laevis*; *Prosorhynchus freitasi* Nagaty, 1937 in *Plectropomus leopardus* and *Plectropomus laevis*; *Prosorhynchus robertsthomsoni* Bott & Cribb, 2009 in *Cephalopholis argus*; *Prosorhynchus longisaccatus* Durio & Manter, 1968 in *Cephalopholis urodeta*, *Epinephelus areolatus*, *Epinephelus cyanopodus* and *Epinephelus maculatus*. *Prosorhynchus luzonicus* Velasquez, 1959 and *Prosorhynchus* sp. B. in *Epinephelus coioides*; *Prosorhynchus serrani* Durio & Manter, 1968 in *Variola albimarginata* and *Variola louti*; *Prosorhynchus* sp. A in *Epinephelus morrhua*; *Prosorhynchus* sp. immature in *Epinephelus coeruleopunctatus*. The new combination *Neidhartia longivesicula* (Bilqees, Khalil, Khan, Perveen & Muti-ur-Rehman, 2009) (Syn. *Prosorhynchus longivesicula*) is formed. Evidence from this paper and earlier molecular studies indicates that there are numerous morphologically similar prosorhynchine species in serranids, most of which show a high degree of host-specificity.

## Abstract (fr)

De nombreuses espèces de Bucephalidae, principalement de la sous-famille Prosorhynchinae, ont été décrites de Serranidae Epinephelinae (mérous) à travers les océans du monde. Dans cet article, huit espèces nommées de Prosorhynchinae sont décrites et / ou illustrées d’Epinephelinae de Nouvelle-Calédonie. *Neidhartia lochepintade* n. sp., parasite d’*Epinephelus chlorostigma*, diffère des autres espèces de *Neidhartia* par des combinaisons variées de taille du corps, taille du rhynchus, distance pré-vitelline et pré-bouche, distance post-testiculaire, étendue du sac du cirre et taille des œufs. Les autres espèces sont : *Neidhartia haywardi* Bott, Miller & Cribb, 2013 chez *Plectropomus leopardus; Neidhartia tyleri* Bott, Miller & Cribb, 2013 chez *Plectropomus leopardus* et *Plectropomus laevis; Prosorhynchus freitasi* Nagaty, 1937 chez *Plectropomus leopardus* et *Plectropomus laevis; Prosorhynchus robertsthomsoni* Bott & Cribb, 2009 chez *Cephalopholis argus; Prosorhynchus longisaccatus* Durio & Manter, 1968 chez *Cephalopholis urodeta*, *Epinephelus areolatus*, *Epinephelus cyanopodus* et *Epinephelus maculatus; Prosorhynchus luzonicus* Velasquez, 1959 et *Prosorhynchus* sp. B. chez *Epinephelus coioides; Prosorhynchus serrani* Durio & Manter, 1968 chez *Variola albimarginata* et *Variola louti; Prosorhynchus* sp. A chez *Epinephelus morrhua; Prosorhynchus* sp. immature chez *Epinephelus coeruleopunctatus*. La nouvelle combinaison *Neidhartia longivesicula* (Bilqees, Khalil, Khan, Perveen & Muti-ur-Rehman, 2009) (Syn. *Prosorhynchus longivesicula*) est formée. Cet article et des études moléculaires antérieures indiquent qu’il existe de nombreuses espèces de Prosorhynchinae morphologiquement semblables chez les Serranidae, dont la plupart présentent un haut degré de spécificité à l’hôte.

## Introduction

Bucephalid digeneans are frequently found in fishes of the family Serranidae, in particular in members of the subfamily Epinephelinae [8]. For example, Bray & Justine [5] listed 16 species of *Prosorhynchus* Odhner, 1905 from serranid fishes: *P. atlanticus* Manter, 1940, *P. bulbosus* Kohn, 1961, *P. caudovatus* Manter, 1940, *P. chorinemi* Yamaguti, 1952, *P. epinepheli* Yamaguti, 1939, *P. freitasi* Nagaty, 1937, *P. gonoderus* Manter, 1940, *P. jupe* (Kohn, 1967), *P. longisaccatus* Durio & Manter, 1968, *P. mcintoshi* (Velasquez, 1959) (this may belong to *Neidhartia*), *P. ozakii* Manter, 1934, *P. pacificus* Manter, 1940, *P. platycephali* (Yamaguti, 1934), *P. promicropsi* Manter, 1940, *P. serrani* Durio & Manter, 1968, and *P. thapari* Manter, 1953, and added a further species *P. maternus* Bray & Justine, 2006. Two were missed, namely *P. aguayoi* Vigueras, 1955 and *P. rarus* (Kohn, 1970). Later, Bott & Cribb [2] added a further five species, *P. jexi* Bott & Cribb, 2009, *P. lafii* Bott & Cribb, 2009, *P. robertsthomsoni* Bott & Cribb, 2009, *P. conorjonesi* Bott & Cribb, 2009 and *P. milleri* Bott & Cribb, 2009 and recently Bott et al. [3] added yet another five species, all from *Plectropomus* spp., *P. lesteri* Bott, Miller & Cribb, 2013, *P. wrightae* Bott, Miller & Cribb, 2013, *P. heronensis* Bott, Miller & Cribb, 2013, *P. munozae* Bott, Miller & Cribb, 2013 and *P. plectropomi* Bott, Miller & Cribb, 2013, making a total of 29 species. Other genera of bucephalids are also reported in serranids, e.g., *Neidhartia* Nagaty, 1937 (*N. neidharti* Nagaty, 1937, *N. ghardagae* Nagaty, 1937, *N. coronata* Durio & Manter, 1968, *N. epinepheli* Bott & Cribb, 2009, *N. tyleri* Bott, Miller & Cribb, 2013, *N. haywardi* Bott, Miller & Cribb, 2013, *N. plectropomi* Bott, Miller & Cribb, 2013), *Pseudoprosorhynchus* Yamaguti, 1938 (*P. hainanensis* Shen, 1990), *Rhipidocotyle* Diesing, 1858 (*R. angusticolle* Chandler, 1941, *R. clavivesiculum* Ku & Shen, 1975), *Bucephalus* Baer, 1827 (*B. heterotentaculatus* Bravo-Hollis & Lamothe-Argumedo, 1956), *Myorhynchus* Durio & Manter, 1968 (*M. pritchardae* Durio & Manter, 1968), *Muraenicola* Nolan & Cribb, 2010 (syn: *Folliculovarium* Gu & Shen, 1983 pre-occupied) (*M. xishaensis* (Gu & Shen, 1983)), *Neoprosorhynchus* Dayal, 1948 (*N. purius* Dayal, 1948) and *Telorhynchus* Crowcroft, 1947 (*T. arripidis* Crowcroft, 1947).

Most of these species belong to the subfamily Prosorhynchinae Nicoll, 1914, but *Bucephalus* and *Rhipidocotyle* are in the Bucephalinae. These may be accidental records. The only bucephaline species originally described from a serranid is *R. clavivesiculus* which, according to the original description [22], has a recurved pars prostatica and sperm duct, a characteristic of the Prosorhynchinae [33].

This paper expands on the records made in Justine et al. [19], discussing the systematics of the reports in that paper, and adding new data obtained subsequently.

## Materials and methods

Digeneans were collected live, immediately fixed in nearly boiling saline and then transferred to 80% ethanol. Whole mounts were stained with Mayer’s paracarmine, cleared in beechwood creosote and mounted in Canada balsam. Measurements were made through a drawing tube on an Olympus BH-2 microscope, using a Digicad Plus digitising tablet and Carl Zeiss KS100 software adapted by Imaging Associates, and are quoted in micrometres. The following abbreviations are used: BMNH, British Museum (Natural History) Collection at the Natural History Museum, London, UK; MNHN JNC, Muséum National d’Histoire Naturelle, Paris, France.

Use has been made of the visual key to *Prosorhynchus* developed by Bray & Palm [6]. (http://www.nhm.ac.uk/bray2009) and a similar key to the genus *Neidhartia* recently devised by us. We use the term “cirrus-sac reach” for the distance from the anterior-most extremity of the cirrus-sac to the posterior extremity of the body as a percentage of the body-length.

## Results

Family Bucephalidae Poche, 1907

Subfamily Prosorhynchinae Nicoll, 1914

Genus *Neidhartia* Nagaty, 1937

urn:lsid:zoobank.org:act:380959E0-57F5-44FB-87FE-EB7B4958CCB6

### *Neidhartia lochepintade* n. sp. (Figures 1, 2)

Syn. *Prosorhynchus* sp. in *Epinephelus chlorostigma* of Justine et al. (2010).

**Figure 1–6.**
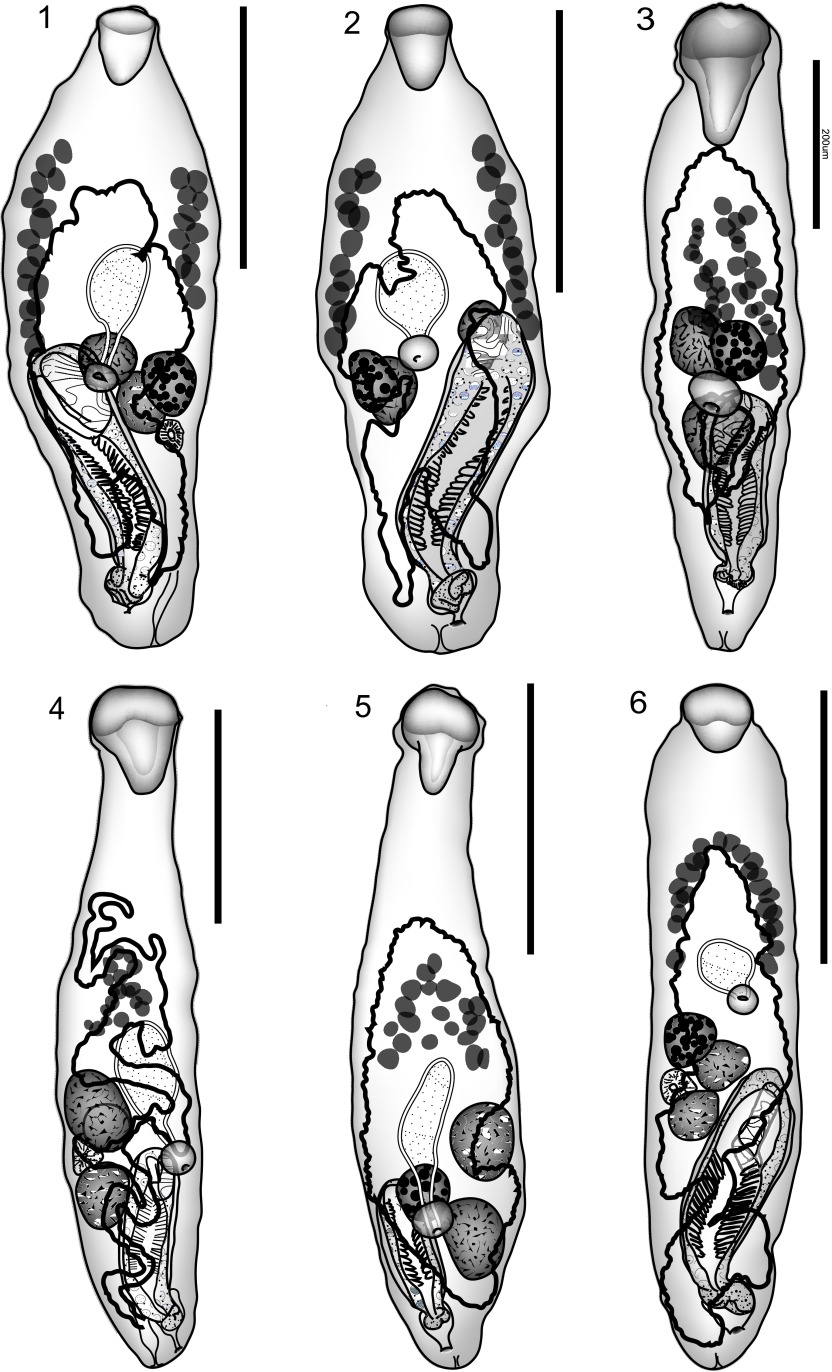
1: *Neidhartia lochepintade* n. sp. Holotype, uterus in outline. 2: *Neidhartia lochepintade* n. sp. Paratype, uterus in outline. 3: *Neidhartia haywardi* Bott, Miller & Cribb, 2013, uterus in outline. 4: *Neidhartia tyleri* Bott, Miller & Cribb, 2013 ex *Plectropomus leopardus*, uterus in outline. 5: *Neidhartia tyleri* Bott, Miller & Cribb, 2013, ex *Plectropomus laevis*, uterus in outline. 6: *Prosorhynchus robertsthomsoni* Bott & Cribb, 2009. Ventral view, uterus in outline. Scale bars: 500 μm (Figs. 1, 2, 4–6); 200 μm (Fig. 3).

urn:lsid:zoobank.org:act:A3A03B8A-A686-4168-AFE5-5F6A54C923BA

Type-Host: *Epinephelus chlorostigma* (Valenciennes) brown-spotted grouper, Serranidae.

Site: Pyloric caeca.

Type-Locality: Off Récif Toombo, deep-sea (22°34′431S, 166°27′552E, 04/01/2008);

Other locality: Off Récif Toombo, deep-sea, 200–300m (22°34′187S, 166°26′292E, 01/12/2009).

Prevalence: 67% (2 of 3).

Type-specimens: Holotype MNHN JNC 2446d-1, Paratypes, MNHN JNC 2446d-2-5, JNC 3141, BMNH 2013.11.18.1.

Etymology: Loche Pintade is the New Caledonian name for the host species.

#### Description

Based on 10 whole-mount preparations. Measurements and ratios in Table 1. Body fusiform, widest at about mid-body (Figures 1, 2). Tegument spinous; spines squamous, tiny, reach to posterior extremity. Rhynchus broad, relatively short and blunt. Mouth at about level of ovary, distinctly in post-equatorial half of body. Pharynx small, globular. Caecum oval, directed anteriorly.

**Table 1.** Measurements and ratios of *Neidhartia* spp. % refers to % of body-length.

Species	*Neidhartia lochepintade* n. sp.			*Neidhartia haywardi*			*Neidhartia tyleri*			*Neidhartia tyleri*		
Host	*Epinephelus chlorostigma*			*Plectropomus leopardus*			*Plectropomus leopardus*			*Plectropomus laevis*		
*n*	10			5			7			6		
	min.	max.	mean	min.	max.	mean	min.	max.	mean	min.	max.	mean
Length	938	1,252	1,159	658	744	715	1,031	1,663	1,392	1,204	1,512	1,328
Width	299	460	391	133	209	176	282	355	319	240	396	333
Previtelline distance	193	279	232	185	252	224	442	671	527	310	602	452
Precaecal distance	311	478	428	336	336	336	525	791	646	634	914	756
Pre-uterine distance	259	361	312	168	222	186	217	487	384	262	518	383
Pre-mouth distance	497	734	655	470	472	471	730	1,273	995	903	1,263	1,028
Pretesticular distance	422	588	541	295	385	348	547	1,082	836	565	875	726
Pre-ovarian distance	451	634	596	311	396	368	614	1,070	862	649	1,115	859
Rhynchus length	114	163	145	145	178	165	207	255	225	189	234	218
Rhynchus width	96	132	109	114	133	125	143	216	174	145	198	166
Rhynchus to vitellarium distance	35	235	102	32	93	64	226	426	303	74	387	229
Rhynchus to uterus distance	127	357	183	1	35	21	8	276	163	32	312	161
Rhynchus to caecum distance	196	443	308	152	152	152	317	530	427	408	684	538
Long vitelline field	312	465	375	104	236	149	160	465	344	192	485	297
Short vitelline field	256	511	339	98	142	114	104	355	230	130	336	246
Caecum length	133	203	165	109	109	109	117	279	186	170	260	217
Caecum width	94	131	112	55	55	55	86	133	100	69	86	80
Pharynx length	52	74	65	48	59	54	42	82	72	66	82	73
Pharynx width	59	74	65	58	64	61	53	94	76	67	84	77
Ovary length	98	122	111	62	85	71	73	173	118	105	136	120
Ovary width	77	110	97	50	68	60	79	138	110	99	123	112
Distance between ovary and anterior testis	0	25	3	0	58	12	0	27	4	0	71	26
Anterior testis length	84	128	107	55	91	75	115	179	150	147	217	175
Anterior testis width	75	116	92	54	73	63	116	168	142	130	194	155
Distance between testes	31	109	76	0	72	24	0	29	11	36	80	56
Posterior testis length	89	127	108	72	87	81	114	195	154	159	217	180
Posterior testis width	75	114	89	43	82	63	103	158	130	123	171	142
Posterior testis to cirrus-sac	0	0	0	0	0	0	0	0	0	0	0	0
Cirrus-sac length	363	595	480	179	217	202	217	360	311	239	353	308
Cirrus-sac width	117	169	141	66	94	79	91	148	128	99	116	109
Seminal vesicle length	146	164	158	63	119	78	79	179	118	73	148	112
Seminal vesicle width	70	117	88	37	54	45	40	115	65	41	88	64
Pars prostatica length	459	573	515	240	333	284	295	416	361	0	503	299
Pars prostatica width	64	95	81	51	69	61	59	109	79	61	85	74
Post-testicular distance	322	479	396	193	231	207	217	336	256	124	214	158
Post-vitelline distance	428	685	525	291	369	332	286	656	498	468	644	565
Cirrus-sac reach	490	637	564	278	346	312	322	510	444	355	491	432
Post-ovarian distance	402	463	431	249	303	282	317	502	407	269	473	335
Post-genital pore distance	37	63	50	30	48	42	30	81	47	29	57	41
Egg length	26	32	30	22	25	23	23	31	28	34	39	37
Egg width	13	22	17	11	15	13	15	20	18	16	19	17
Width %	30.1	38.1	33.7	20.2	28.5	24.5	19.3	32.4	23.6	19.2	28.9	25.1
Previtelline distance %	16.0	22.6	20.1	28.1	34.1	31.2	29.5	51.2	38.5	25.7	43.6	33.8
Precaecal distance %	33.1	38.8	36.9	45.4	45.4	45.4	48.4	50.9	49.9	52.7	60.4	55.3
Pre-uterine distance %	23.0	29.4	26.9	22.6	30.0	26.0	21.0	35.9	27.3	21.8	37.5	28.7
Pre-mouth distance %	52.9	60.0	56.7	63.2	63.8	63.5	66.2	76.5	72.6	73.9	83.6	77.3
Pretesticular distance %	44.9	51.3	47.7	44.9	52.1	48.5	53.0	65.0	59.5	46.9	60.8	54.4
Pre-ovarian distance %	48.0	54.2	51.8	47.3	54.3	51.4	58.9	64.4	61.8	51.4	73.8	64.3
Rhynchus length %	11.4	13.4	12.5	19.9	24.1	23.0	14.6	20.9	16.5	13.7	19.4	16.5
Rhynchus width % rhynchus length	63.2	101	75.8	66.9	81.2	76.0	63.0	105	77.8	66.3	105	76.9
Longest vitelline field %	28.1	37.1	32.4	15.8	31.7	20.8	15.6	31.7	24.2	15.3	32.1	22.1
Caecal length %	11.9	16.3	14.2	14.8	14.8	14.8	10.6	19.0	13.9	14.1	17.9	15.9
Ovary length %	8.76	10.5	9.7	8.62	12.1	9.97	6.55	11.1	8.50	8.01	10.8	9.07
Anterior testis length %	8.25	11.1	9.4	8.37	12.4	10.5	8.84	12.5	10.8	10.9	18.0	13.3
Distance between testes %	3.36	9.43	6.62	0	9.70	3.27	0	1.96	0.79	2.58	6.33	4.33
Posterior testis length %	7.38	11.7	9.7	10.9	12.0	11.4	9.37	13.3	11.1	11.7	16.0	13.6
Posterior testis to cirrus-sac %	0	0	0	0	0	0	0	0	0	0	0	0
Cirrus-sac length %	30.4	52.9	41.8	24.2	31.6	28.4	20.6	26.8	22.4	19.1	27.4	23.3
Seminal vesicle length % of cirrus-sac length	27.2	35.1	29.9	30.7	57.0	39.1	29.6	49.7	36.0	28.5	44.4	35.8
Post-testicular distance %	30.2	39.7	34.7	26.1	35.1	29.0	14.9	24.0	18.6	9.2	16.9	12.1
Post-vitelline distance %	39.7	56.8	45.3	39.1	56.1	46.7	27.7	42.1	35.5	33.9	51.0	43.0
Cirrus-sac reach %	41.1	59.2	49.0	37.6	52.7	43.8	30.1	34.5	32.0	28.3	38.9	32.7
Post-ovarian distance %	35.3	43.0	37.6	35.3	45.5	39.5	27.2	33.4	29.4	17.8	37.4	25.6
Post-genital pore distance %	3.04	6.70	4.41	4.10	6.86	5.96	1.97	5.45	3.47	1.92	4.52	3.13

Testes 2, irregularly oval, oblique, in about mid-body, usually well separated. Cirrus-sac elongate, more-or-less parallel sided, reaching anterior testis, anteriorly to pharynx. Seminal vesicle elongate-oval, in proximal cirrus-sac. Pars prostatica long, in two distinct parts; proximal part narrow, coiled over seminal vesicle; distal part wider, straighter, surrounded by dense layer of gland-cells, lining of filaments in chevron arrangement. Ejaculatory duct narrow, opening on large, complex genital lobe inside genital atrium. Genital atrium large. Genital pore distinctly separated from posterior extremity.

Ovary oval, intertesticular, overlapping posterior testis. Mehlis’ gland overlapping ovary and posterior testis. Details of proximal female system obscured by eggs. Uterus not reaching anteriorly to vitelline fields, fills most of available space to level of genital pore. Eggs numerous, tanned, operculate. Metraterm not detected, obscured by eggs. Vitellarium consists of two lateral fields of 12–15 follicles, more or less symmetrical, but with one field slightly longer than other, anterior extremity distinctly posterior to rhynchus and anterior to uterus, always anterior to caecum and gonads; posterior extremity at about level of ovary.

Excretory pore terminal; anterior extent of vesicle obscured by eggs.

#### Discussion

The features that distinguish *N. lochepintade* from previously described *Neidhartia* species are discussed below; comparative metrical data in Table 2.

**Table 2.** Comparisons of *Neidhartia* spp., blue shading shows major distinctions, green shading shows minor distinctions.

Species	Length μm	Width %	Rhynchus length %	Previtelline distance %	Pre-uterine distance %	Pre-mouth distance %	Post-testicular distance %	Cirrus-sac reach %	Egg-size μm	Source
*Neidhartia lochepintade* n. sp.	1,067–1,252	30–38	11–13	16–23	23–29	53–60	30–40	41–59	26–32 × 13–19	new data
*Neidhartia haywardi*	**658–744**	20–28	**20–24**	**28–34**	23–30	**63–64**	26–35	38–53	**22–25 × 11–15**	new data
*Neidhartia haywardi*	731–1,073	23–28	**18–26**	?	31	66	**18**	31	20–23 × 11–13	[3]
*Neidhartia plectropomi*	700–1,245	11–26	**17–24**	?	28	67	21	31	28–33 × 16–20	[3]
*Neidhartia tyleri* ex *P. leopardus*	1,031–1,663	**19–32**	**15–21**	**29–51**	21–36	66–77	**15–24**	**30–35**	23–31 × 15–20	new data
*Neidhartia tyleri* ex *P. laevis*	1,204–1,512	**19–29**	**14–19**	26–44	22–38	**74–84**	**9–17**	**28–39**	**34–39 × 16–19**	new data
*Neidhartia tyleri*	1,203–1,544	**16–22**	**14–20**	**42**	30	**76**	**18**	**30**	**38–44 × 22–26**	[3]
*Neidhartia coronata*	1,392–1,949	**14–15**	**17**	**47**	**52**	**81**	**15**	**17**	**33–38 × 17–22**	[9]
*Neidhartia epinepheli*	880–896	25	**18–19**	**35**	**14**	**65**	**25**	**36**	25–26 × 13	[3]
*Neidhartia ghardagae*	561–908	24–27	**21–37**	**34–40**	29–33	**77**	**21–23**	**29–33**	31 × 20	[27]
*Neidhartia longivesicula*	**1,910–2,120**	33–36	15–16	12	20	**34**	45	44	29–31 × 11–12	[1]
*Neidhartia microrhyncha*	**1,390–2,930**	**14–17**	8–10	27	?	59	33	**19**	none	[7]
*Neidhartia mcintoshi*	820–1,000	26–30	**14–21**	23	**56**	**48**	29	**31**	26–34 × 17–26	[45]
*Neidhartia neidharti*	842–2,112	11–29	**20–27**	24	21	**74**	**21**	**36**	19–29 × 15–19	[27]
*Neidhartia polydactyli*	1,415	**16**	16	**41**	28	**78**	30	38	30–32 × 21–22	[25]
*Pseudoprosorhynchus hainansis*	**2,552**	**21**	9	26	17	57	33	**31**	**21–24 × 12–13**	[39]

*Neidhartia coronata* Durio & Manter, 1968, based on “six somewhat macerated, extended specimens” from the intestine of a “Serranidae”, “probably *Epinephelus*”, from off New Caledonia [9], is narrower, with a larger rhynchus, longer previtelline distance, longer pre-uterine distance, longer pre-mouth distance, shorter post-testicular distance, shorter cirrus-sac reach and greater egg-size. The host identifications in Durio & Manter [9] are often rather vague, and this case is no exception. In this particular case, Durio & Manter’s “*Epinephelus*” could be any Serranidae, including any species of *Cephalopholis*, *Plectropomus*, *Variola* and even *Epinephelus*.

*Neidhartia epinepheli* Bott & Cribb, 2009, based on two specimens from the intestine of the highfin grouper *Epinephelus maculatus* (Bloch) (Serranidae) off Lizard Island on the Great Barrier Reef [2], has a relatively larger rhynchus and a longer previtelline distance. In *N. epinepheli* the uterus reaches anterior to the vitellarium. Other probably differences are the pre-mouth distance, post-testicular region and cirrus-sac reach.

*Neidhartia ghardagae* Nagaty, 1937, based on 16 specimens from a “*Serranus* sp.” from off Ghardaga in the Red Sea [29], has a relative larger rhynchus, longer previtelline distance and longer pre-mouth distance and probably a shorter post-testicular region and a shorter cirrus-sac reach.

*Neidhartia haywardi* Bott, Miller & Cribb, 2013, based on 10 specimens and ITS2 sequence from *Plectropomus leopardus*, *P. laevis* and the spotted coralgrouper *P. maculatus* (Bloch), from Heron and Lizard Islands on the Great Barrier Reef [3] has a bigger rhynchus, longer previtelline distance and shorter post-testicular distance.

*Neidhartia longivesicula* (Bilqees, Khalil, Khan, Perveen & Muti-ur-Rehman, 2009) n. comb. (Syn. *Prosorhynchus longivesicula*) is based on seven specimens from the yellow-tail scad *Atule mate* (Cuvier) (as *Caranx affinus* Rüppell) (Carangidae) off Karachi in the northern Arabian Sea [1]. The ovary is described as “posterior to anterior testis and ventro-lateral to posterior testis”, indicating that the species belongs to *Neidhartia*. This species differs from *N. lochepintade* particularly in the more anterior mouth and greater body-size.

*Neidhartia mcintoshi* Velasquez, 1959, based on two mature and four immature specimens from the muscle, stomach and intestine of the duskytail grouper *Epinephelus bleekeri* (Vaillant) (Serranidae) off Malabon, Rizal, Luzon Island, Philippines [48], has a longer pre-uterine extent, and probably a relatively larger rhynchus, shorter pre-mouth distance and shorter cirrus-sac reach. In connection with unusual sites of infection given, Velasquez [48] stated that the “present species occurs as metacercaria and adult in the same host, showing evidence that infection of one fish is brought about possibly through the eating of the smaller fish by the larger”.

*Neidhartia microrhyncha* Chauhan, 1943, based on five non-ovigerous specimens from the alimentary canal of the Indian spiny turbot *Psettodes erumei* (Bloch & Schneider) (Psettodidae) off Bombay (now Mumbai), India [7], is narrower and has a shorter cirrus-sac reach. It is reported to grow much bigger.

*Neidhartia neidharti* Nagaty, 1937, based on eight specimens from *Serranus* sp. locally called “Nagil”, from off Ghardaga in the Red Sea [29], has a relatively larger rhynchus and longer pre-mouth distance and probably a shorter post-testicular region and a shorter cirrus-sac reach. The vitellarium overlaps the rhynchus. According to Froese & Pauly [15] the common name “Nagil” refers to either the squaretail coralgrouper *Plectropomus areolatus* (Rüppell, 1830) or the roving coralgrouper *Plectropomus pessuliferus* (Fowler, 1904) (Serranidae).

*Neidhartia plectropomi* Bott, Miller & Cribb, 2013 based on 10 specimens and ITS2 sequence from *Plectropomus leopardus* and *P. laevis* from Heron and Lizard Islands on the Great Barrier Reef [3] has a bigger rhynchus and longer previtelline distance.

*Neidhartia polydactyli* Manter, 1953, based on a single specimen from the intestine of the striped threadfin *Polydactylus plebeius* (Broussonet) (Polynemidae) off Suva, Fiji [26], has a relatively larger rhynchus and longer previtelline and pre-mouth distances.

*Neidhartia tyleri* Bott, Miller & Cribb, 2013 based on 10 specimens and ITS2 sequence from the *Plectropomus leopardus*, *P. laevis* and *P. maculatus*, from Heron and Lizard Islands on the Great Barrier Reef [3] is narrower, with longer previtelline and pre-mouth distances, shorter post-testicular distance and cirrus-sac reach, and larger eggs.

*Pseudoprosorhynchus hainansis* Shen, 1990, based on two specimens from the intestine of the *Plectropomus leopardus* off Hainan Island, southern China [41] is similar to *Neidhartia lochepintade* (and indeed the whole genus) in that the ovary is between the testes, but the rhynchus is disc-like, and the worm is long and narrow. It also appears to have a short cirrus-sac reach and smaller eggs.

These data, and the record from this deep-water serranid, indicate to us that the specimens described here belong to a new species. *Prosorhynchus epinepheli* Yamaguti, 1939 has been reported twice from this host, from off Tuticorin, India [18] and from the Arabian Gulf [40]. The illustrations in both papers show that the ovary lies partly anterior to and partly overlapping the anterior testis, and thus do not indicate that the worm in question is a *Neidhartia*. The Indian record [18] is from several host species and it is not stated from which the illustrated worm was collected. *E. chlorostigma* has also been listed as a host for unnamed *Prosorhynchus* spp. in the Arabian Gulf [11, 39].

As discussed below, the generic status of *Prosorhynchus epinepheli* and *P. longisaccatus* is ambiguous as often the ovary does not lie distinctly anteriorly to the testes, suggesting that they may be *Neidhartia* spp. Comparison of data in Tables 2 and 6 indicates that the rhynchus is relatively much larger in *P. epinepheli* and *P. longisaccatus*. The pre-uterine distance tends to be larger in *P. longisaccatus*, but overlaps considerably.

### *Neidhartia haywardi* Bott, Miller & Cribb, 2013 (Figure 3)

urn:lsid:zoobank.org:act:47F33650-B6E4-414C-9F58-320F4F05E504

Host: *Plectropomus leopardus* (Lacepède) (Perciformes: Serranidae), leopard coralgrouper.

Site: digestive tract

Localities: Grande Rade, Nouméa 22°15′S 166°24E, 23/10/2007 and 24/10/2007; Between Larégnière and Récif Crouy, 22°20′702S, 166°19′295E, 05/05/2008.

Prevalence: 57% (4 of 7).

Vouchers: MNHN JNC2333B, JNC2333C, JNC2334, JNC2513; BMNH 2013.11.18.5-6.

#### Description

Based on five whole-mount preparations. Measurements and ratios in Table 1. Body widest at about mid-body (Figure 3). Tegument spinous; spines squamous, tiny, reach to posterior extremity. Rhynchus broad, conical or bluntly conical. Mouth just posterior to ovary, well into post-equatorial half of body. Pharynx small, globular. Caecum oval, directed anteriorly.

Testes 2, irregularly oval, oblique, in about mid-body, usually well separated. Cirrus-sac elongate, more-or-less parallel sided, reaching to or almost to anterior testis, anteriorly to pharynx. Seminal vesicle elongate-oval, in proximal cirrus-sac. Pars prostatica long, in two distinct parts; proximal part narrow, coiled over seminal vesicle; distal part, wider, straighter, surrounded by dense layer of gland-cells, lining of filaments in chevron arrangement. Ejaculatory duct narrow, opening on large, complex genital lobe, inside genital atrium. Genital atrium large. Genital pore distinctly separated from posterior extremity.

Ovary oval, intertesticular, overlapping testes. Mehlis’ gland overlapping ovary and posterior testis. Details of proximal female system obscured by eggs. Uterus reaches anteriorly to vitelline fields, occasionally to level of vitellarium, fills much of available space to level of genital pore. Eggs numerous, tanned, operculate. Metraterm not detected, obscured by eggs. Vitellarium consists of two lateral fields of 12–15 follicles, more or less symmetrical, but with one field slightly longer than other, anterior extremity posterior to rhynchus and anterior extent uterus, reaches anterior to caecum and gonads; posterior extremity at about level of ovary.

Excretory pore terminal; anterior extent of vesicle obscured by eggs.

#### Discussion

This form appears to be *N. haywardi* or *N. plectropomi* differing only in the previtelline distance, as calculated from the illustration [3, Figure 3], but it should be noted that in both species Bott et al. [3] found that the extent of the vitellarium was obscured by the uterus. *N. haywardi* and *N. plectropomi* are sister species according to the molecular study of Bott et al. [3]. We consider our specimens to be *P. haywardi* as the egg-sizes more nearly coincide (Table 2), but the cirrus-sac reach of our specimens tends to be greater than is apparent in either species. Both *P. haywardi* and *P. plectropomi* are reported from *P. leopardus* and *P. laevis*, and from Heron and Lizard Islands on the Great Barrier Reef.

The features distinguishing this species from its congeners can be seen in Table 2, and two further species are not easily distinguished, namely *N. neidharti* Nagaty, 1937 and *N. epinepheli* Bott & Cribb, 2009.

*N. neidharti* was first reported in *Serranus* sp. locally known as “Nagil” from the Red Sea [29]. According to Froese & Pauly [15] this common name refers to the squaretail coralgrouper *Plectropomus areolatus* (Rüppell) or the roving coralgrouper *P. pessuliferus* (Fowler). It seems clear, therefore, that it is a parasite of *Plectropomus*. Chauhan [7] recorded, but did not describe, this species in *Belone* sp. (Beloniformes: Belonidae) from Mumbai (Bombay), India. As unlikely as this combination of hosts is, its putative hosts associations become even more puzzling when the record by Maurya et al. [27] in the freshwater long-whiskered catfish *Sperata* (= *Mystus*) *aor* (Hamilton) (Siluriformes: Bagridae) from Uttar Pradesh, India is considered. We are discounting the Indian records of this species. *N. neidharti* apparently grows to a much greater size than *N. plectropomi*, although there is room for confusion. In Nagaty’s [29] description (p. 119) the length range is given as 561–908, whereas in the table of measurements (p. 166) the length is given as 842–2,112 (vs. 658–744 (715) for *P. haywardi*). This confusion also applies to width where, using the data from the description, the range is 24–27% and in the table it is 11–29% of body-length (vs. 20–24%). The body-width in Nagaty’s Figure 56 is about 24% of the body-length. The pre-mouth distance may be greater than in *N. haywardi.*

*Neidhartia epinepheli*. Bott & Cribb stated that it “It bears a superficial resemblance to the type-species, *N. neidharti* Nagaty, 1937, in that its uterus extends past the posterior margin of the rhynchus. *N. epinepheli* differs by having a caecum that does not extend into the anterior third of the body and the eggs are smaller, 25–26 × 12–13, compared with 30 × 15 for *N. neidharti* (see Nagaty, 1937)”. The confusion in the egg-size as given by Nagaty [29] for *N. neidharti*, in that he gives the egg-size as 30 × 15 in the text, but 19–29 × 15–19 in the table may well invalidate one of Bott & Cribb’s [2] distinctions. The other distinction is rather minor and it may be found that these species are synonymous. The pre-uterine distance is shorter than in *N. haywardi* in that the uterus overlaps the rhynchus.

### *Neidhartia tyleri* Bott, Miller & Cribb, 2013 (Figures 4, 5)

urn:lsid:zoobank.org:act:E131C73F-7D32-4B80-8656-EB8411FAAE8B

Hosts: *Plectropomus leopardus* (Lacepède) (Perciformes: Serranidae), leopard coralgrouper; *Plectropomus laevis* (Lacepède), blacksaddled coralgrouper.

Site: digestive tract

Localities: (*P. leopardus* & *P. laevis*) Off Ouano (21°49′430S, 166°44′278E, 25/10/2007), *P. leopardus* Near Récif Toombo (22°34′107S, 166°28′816E, 30/09/2009).

Prevalences: *P. leopardus*, 29% (2 of 7), *P. laevis*, 50% (1 of 2).

Vouchers: (*P. leopardus*) MNHN JNC2340, JNC 3060B; BMNH 2013.11.18.2-3; (*P. laevis*) JNC2339; BMNH 2013.11.18.4.

#### Description

Based on seven whole-mount preparations from *P. leopardus* and six from *P. laevis*. Measurements and ratios in Table 1. Body fusiform, widest in posterior third (Figures 4, 5). Tegument spinous; spines squamous, tiny, reach to posterior extremity. Rhynchus broad, with narrow conical posterior extension. Mouth at about level of ovary or just posterior, well into post-equatorial half of body. Pharynx small, globular. Caecum elongate-oval, directed anteriorly.

Testes 2, irregularly oval, oblique to tandem, in about mid-body, slightly separated or not. Cirrus-sac elongate, more-or-less parallel sided, reaching anterior testis, anteriorly to pharynx. Seminal vesicle elongate-oval, in proximal cirrus-sac. Pars prostatica long, in two distinct parts; proximal part narrow, coiled over seminal vesicle; distal part, wider, straighter, surrounded by dense layer of gland-cells, lining of filaments in chevron arrangement. Ejaculatory duct narrow, opening on large, complex genital lobe inside genital atrium. Genital atrium large. Genital pore distinctly separated from posterior extremity.

Ovary oval, intertesticular, overlapping testes. Mehlis’ gland overlapping ovary and posterior testis. Details of proximal female system obscured by eggs. Uterus not reaching anteriorly to vitelline fields, fills much of available space to level of genital pore. Eggs numerous, tanned, operculate. Metraterm not detected, obscured by eggs. Vitellarium consists of two lateral fields of follicles, more or less symmetrical, but with one field slightly longer than other, anterior extremity distinctly posterior to rhynchus and anterior to uterus, always reaches anterior to caecum and gonads; posterior extremity at or just posterior to level of ovary.

Excretory pore terminal; anterior extent of vesicle obscured by eggs.

#### Discussion

We have identified the larger *Neidhartia* specimens as belonging to *N. tyleri*. Most morphological characters are similar (Table 2), but the eggs in our specimens from *P. leopardus* (the type-host of *N. tyleri*) are distinctly smaller than those described for this species [3] and our specimens from *P. laevis*. This species is readily distinguished from most described species (Table 2). *N. neidharti* is not distinguishable from the specimens from *P. laevis* in major features of the visual key and differs from the *P. leopardus* specimens only in rhynchus length (Table 2). This feature probably distinguishes this form from *N. neidharti* as the *P. laevis* specimens do not overlap in this feature. Comparison with *N. neidharti* as described by Nagaty [29] is problematical as the measurements given in the description and table do not coincide, but our specimens are very distinct from the illustrated specimen [28, Figure 56] in shape (relatively more elongate, although the measurements in the table do not bear this out), the previtelline distance and pre-uterine distance.

*N. coronata* Durio & Manter, 1968, described from “Serranidae, probably *Epinephelus* sp.” from off New Caledonia [9], differs from our specimens in the visual key in the pre-uterine distance and cirrus-sac reach. It should be borne in mind, however, that Durio & Manter [9] stated that their description was “based on six somewhat macerated, extended specimens”. The previtelline distance may also be a distinguishing feature.

Genus *Prosorhynchus* Odhner, 1905

urn:lsid:zoobank.org:act:21111289-7672-4028-830D-A37199B68E26

### *Prosorhynchus robertsthomsoni* Bott & Cribb, 2009 (Figure 6)

urn:lsid:zoobank.org:act:0EEE6ED7-01CE-45A0-A2B9-27F32EBC64CC

Host: *Cephalopholis argus* Bloch & Schneider (Perciformes: Serranidae), peacock hind.

Site: digestive tract

Locality: Near Récif Toombo (22°31′30″S, 166°26′40″E, 03/11/2006).

Prevalence: 50% (1 of 2).

Vouchers: MNHN JNC 2110; BMNH 2013.11.18.25.

#### Discussion

Measurements and ratios are given in Table 3. This species is known only from *Cephalopholis argus*, the coral hind *Cephalopholis miniata* (Forsskål) and the bluespotted hind *C. cyanostigma* (Valenciennes) from off Heron and Lizard Islands on the Great Barrier Reef [2, 3]. Using the visual key our specimens align with four species, in addition to *P. robertsthomsoni*. Distinctions are tabulated in Table 4.

**Table 3.** Measurements and ratios of *Prosorhynchus* spp. from *Cephalopholis* spp. % refers to % of body-length.

Species	*Prosorhynchus robertsthomsoni*			*Prosorhynchus longisaccatus*
Host	*Cephalopholis argus*			*Cephalopholis urodeta*
n	7			1
	min.	max.	mean	
Length	1,088	1,256	1,171	836
Width	251	291	270	333
Previtelline distance	192	281	238	192
Precaecal distance	345	474	399	261
Pre-uterine distance	117	298	245	289
Pre-mouth distance	504	577	540	458
Pretesticular distance	390	654	549	326
Pre-ovarian distance	467	676	559	341
Rhynchus length	112	144	130	223
Rhynchus width	97	145	122	166
Rhynchus to vitellarium distance	82	144	108	0
Rhynchus to uterus distance	79	170	131	58
Rhynchus to caecum distance	231	475	309	36
Long vitelline field	247	319	284	288
Short vitelline field	181	264	236	228
Caecum length	84	120	103	138
Caecum width	68	114	96	125
Pharynx length	42	53	48	67
Pharynx width	44	55	51	75
Ovary length	83	98	90	81
Ovary width	75	99	87	62
Distance between ovary and anterior testis	0	0	0	0
Anterior testis length	86	100	93	90
Anterior testis width	81	109	96	81
Distance between testes	0	89	34	30
Posterior testis length	75	107	91	82
Posterior testis width	61	109	88	96
Posterior testis to cirrus-sac	0	0	0	0
Cirrus-sac length	308	417	368	340
Cirrus-sac width	96	141	119	161
Seminal vesicle length	134	185	152	?
Seminal vesicle width	37	59	47	?
Pars prostatica length	346	523	435	?
Pars prostatica width	59	128	95	?
Post-testicular distance	350	460	405	375
Post-vitelline distance	588	713	653	406
Cirrus-sac reach	428	550	505	427
Post-ovarian distance	440	604	512	421
Post-genital pore distance	53	113	74	58
Egg length	32	38	34	30
Egg width	16	20	18	20
Width %	22.5	24.3	23.1	39.8
Previtelline distance %	17.7	23.3	20.3	23.0
Precaecal distance %	31.6	38.0	34.1	31.3
Pre-uterine distance %	10.8	25.7	20.9	34.5
Pre-mouth distance %	44.8	47.5	46.0	54.8
Pretesticular distance %	35.9	52.5	46.7	39.0
Pre-ovarian distance %	42.9	56.0	47.7	40.8
Rhynchus length %	9.95	12.4	11.1	26.7
Rhynchus width % rhynchus length	82.8	106	94.2	74.4
Longest vitelline field %	19.8	26.3	24.3	34.4
Caecal length %	6.7	10.4	8.9	16.4
Ovary length %	7.12	8.51	7.72	9.66
Anterior testis length %	7.23	8.84	7.94	10.8
Distance between testes %	0	8.14	2.96	3.59
Posterior testis %	6.77	9.79	7.81	9.85
Posterior testis to cirrus-sac %	0	0	0	0
Cirrus-sac length %	27.7	33.7	31.4	40.6
Seminal vesicle length % cirrus-sac length	35.0	44.5	39.0	?
Post-testicular distance %	30.9	39.6	34.7	44.9
Post-vitelline distance %	53.0	58.1	55.8	48.6
Cirrus-sac reach %	38.6	48.0	43.1	51.0
Post-ovarian distance %	36.4	48.8	43.7	50.3
Post-genital pore distance %	4.56	10.4	6.28	6.99

**Table 4.** Comparisons of *Prosorhynchus robertsthomsoni*, green shading shows minor distinctions.

Species	Length	Width %	Rhynchus L %	Previtellarium %	Pre-Uterine %	Mouth	Post-testicular %	Cirrus-sac reach %	Eggs	Reference
*P. robertsthomsoni* Bott & Cribb, 2009	1,088–1,256	23–24	10–12	18–23	10–26	45–47	31–40	39–48	32–38 × 16–20	new data
*P. robertsthomsoni* Bott & Cribb, 2009	1,072–1,408	18–23	10–11	27	24	48	31	35	29–30 × 16	[2]
*P. aguayoi* Vigueras, 1955	1,700	**29**	9	21	18	46	**44**	36	40 × 26	[47]
*P. jexi* Bott & Cribb, 2009	1,104–1,424	19–25	15	20	**32**	43	41	37	32–33 × 16	[2]
*P. serrani* Durio & Manter, 1968	**1,027–2,245**	**22**	**12–18**	20	17	**49**	**27**	**33**	24–29 × 15–21	[9]
*P. serrani* Durio & Manter, 1968	816–1,826	30–32	**15–16**	30–34	31	**53–62**	**24–31**	**31–36**	25–29 × 17–21	[27]
*P. tsengi* Tsin, 1933	1,500–1,800	24–25	**7**–**9**	22	19	47	34	41	**19–25 × 14–17**	[44]

*Prosorhynchus aguayoi* Vigueras, 1955 from the greater soapfish *Rypticus saponaceus* (Bloch & Schneider) (Serranidae) from off Cuba, Curaçao and Jamaica [30, 31, 50] is a very similar species to *P. robertsthomsoni* but is probably wider and more fusiform, with a longer post-testicular region. The vitellarium reaches the testes in *P. aguayoi* and the cirrus-sac does not.

*Prosorhynchus jexi* (syn: *P. epinepheli* of Durio &Manter (1968)) from the longfin grouper *Epinephelus quoyanus* (Valenciennes) (Serranidae) from the Great Barrier Reef [2, 9] differs from *P. robertsthomsoni* in the more restricted uterus. Bott & Cribb [2] considered that the reach of the uterus anterior to the vitellarium is a distinctive feature of *P. robertsthomsoni* but our observations indicate that this does not always occur (Figure 6). The cirrus-sac does not reach the testes in *P. jexi*.

*Prosorhynchus serrani* Durio & Manter, 1968 (syn: *Prosorhynchus crucibulus* of Nagaty (1937)) from the yellow-edged lyretail *Variola louti* (Forsskål) (Serranidae) from the Red Sea and off New Caledonia [9, 29] is very similar to *P. robertsthomsoni* but apparently has a distinctly different shaped rhynchus, in that it has a distinct narrow elongate posterior extension in contrast to the blunt rounded posterior of the *P. robertsthomsoni* rhynchus. It may be that the vitellarium reaches slightly more posteriorly in *P. serrani* in that the follicles extend just posterior to the pharynx, rather than just to the pharynx (see below).

*Prosorhynchus tsengi* Tsin, 1933 is a parasite of the bartail flathead *Platycephalus indicus* (Linnaeus) (Platycephalidae) off China [42, 47]. Bray & Palm [6] pointed out that the “original illustration of *P. tsengi* by Tsin [47, Figure 8] shows a lobed rhynchus, apparently with an aperture, and a straight pars prostatica, indicating that the species may in fact belong to the genus *Rhipidocotyle*”. In addition the rhynchus and eggs appear slightly smaller than in *P. robertsthomsoni.*

### *Prosorhynchus longisaccatus* Durio & Manter, 1968 (Figures 7–10)

urn:lsid:zoobank.org:act:FAB66691-C264-4A99-9585-FF7BEDC41316

**Figure 7–12.**
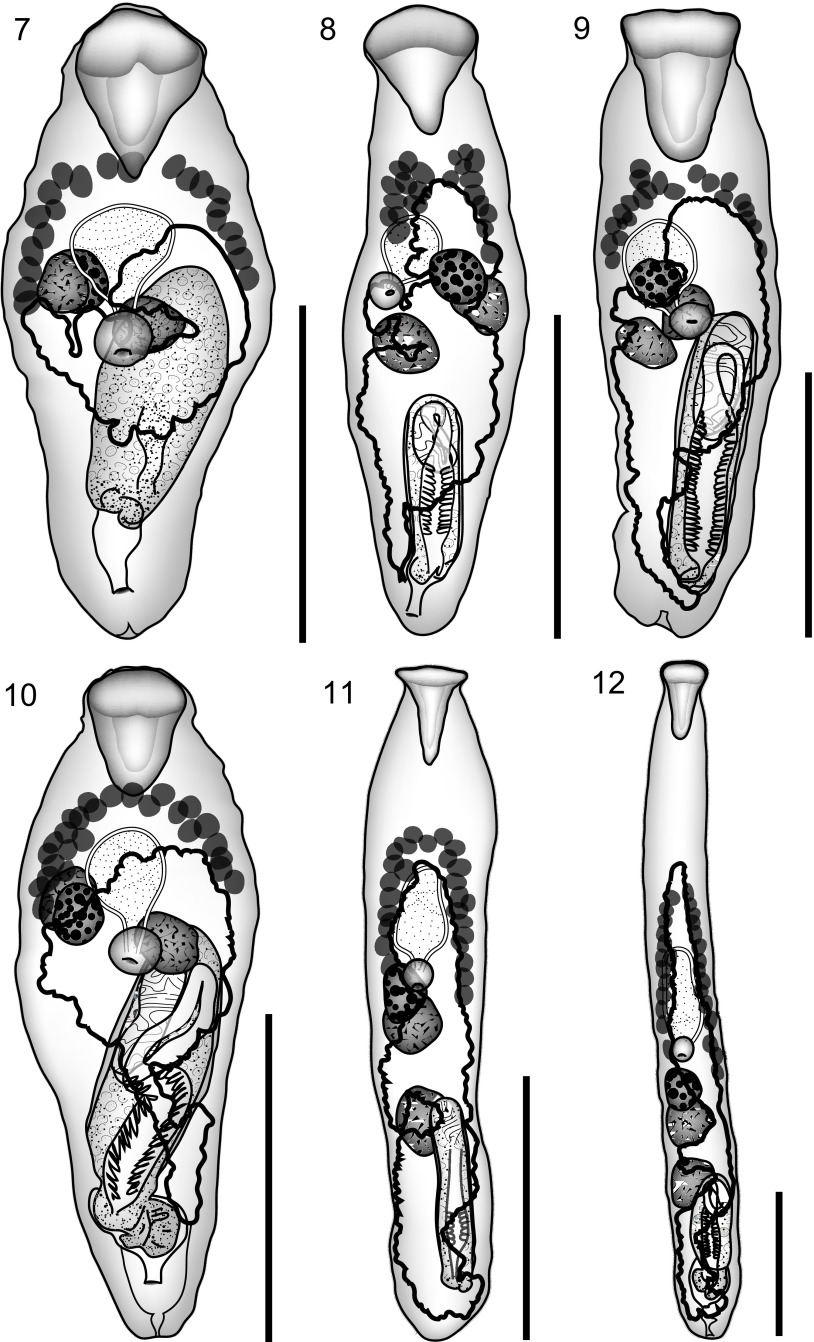
7: *Prosorhynchus longisaccatus* Durio & Manter, 1968 ex *Cephalopholis urodeta.* Ventral view, uterus in outline. 8: *Prosorhynchus longisaccatus* Durio & Manter, 1968 ex *Epinephelus areolatus.* Ventral view, uterus in outline. 9: *Prosorhynchus longisaccatus* Durio & Manter, 1968 ex *Epinephelus cyanopodus.* Ventral view, uterus in outline. 10: *Prosorhynchus longisaccatus* Durio & Manter, 1968 ex *Epinephelus maculatus.* Ventral view, uterus in outline. 11: *Prosorhynchus serrani* Durio & Manter, 1968 ex *Variola albimarginata.* Ventral view, uterus in outline. 12: *Prosorhynchus serrani* Durio & Manter, 1968 ex *Variola louti.* Ventral view, uterus in outline. Scale bars: 500 μm.

Hosts: *Cephalopholis urodeta* (Forster), Serranidae, darkfin hind; *Epinephelus areolatus* (Forsskål), Serranidae, areolate grouper; *Epinephelus cyanopodus* (Richardson), Serranidae, speckled blue grouper; *Epinephelus maculatus* (Bloch), Serranidae, highfin grouper.

Site: Intestine, pyloric caeca, stomach, digestive tract.

Locality : *C. urodeta*, Off Récif Kué, New Caledonia (07/10/2008); *E. areolatus*, Off Pointe Bovis (22°14′S, 166°20′E, 21/10/2008); Nouméa Fish Market (15/06/2007); *E. cyanopodus*, Passe de Dumbéa (22°20′00″S, 166°15′00″E, 25/11/2005 and 05/10/2006), Passe de Boulari (22°30′00S, 166°24′00″E, 05/10/2006), Near Îlot Mato (22°33′E, 166°47′E, 05/08/2007), Baie Maa (22°12′762S, 166°19′924E, 13/11/2007), Baie des Citrons, Nouméa (22°18′S, 166°26′E, 31/03/2009); *E. maculatus*, Phare Amédée (22°27′S, 166°26′E, 20/06/2006), Off Ever Prosperity, external slope, depth 60 m; (22°27′S, 166°21′E, 22/08/2006), Off Ever Prosperity, external slope, depth 60–80 m (22°27′S, 166°21′E, 17/04/2007), Récif Kué, External slope (22°34′892S, 166°29′673E, 19/06/2007), Off Récif Kué (22°36′S, 166°31′E, 07/10/2008), Shallow, Interior Lagoon near Récif Toombo (22°32′583S, 166°28′978E, 05/11/2008), Shallow, Interior Lagoon near Récif Toombo (22°32′536S, 166°29′069E, 20/11/2008), Baie des Citrons, Nouméa (22°18′S, 166°26′E, 09/04/2009), Interior Lagoon near Récif Toombo (22°32′536S, 166°29′069E, 30/04/2009).

Prevalence: *C. urodeta*, 33% (1 of 3); *E. areolatus*, 75% (3 of 4); *E. cyanopodus*, 87% (7 of 8); *E. maculatus*, 61% (16 of 26).

Voucher specimens: *C. urodeta*, MNHN JNC 2683; *E. areolatus*, JNC2690, JNC2691, JNC2175; BMNH 2013.11.18.15-16; *E. cyanopodus*, JNC2270a, JNC1659, JNC1998B, JNC1998C, JNC2000A, JNC2000B, JNC2270, JNC2395, JNC2891A, JNC2891B; BMNH 2006.4.27.1-10, 2013.11.18.22-23; *E. maculatus*, JNC1874, JNC1927, JNC2157D, JNC2187A, JNC 2680, JNC 2754, JNC 2759, JNC2894B, JNC2929, JNC3031, JNC3052, JNC3053, JNC3061, JNC3066, JNC3067; BMNH 2007.5.2.39-41, 2013.11.18.17-21.

#### Description

See Tables 3 and 5 for measurements and ratios based on 52 specimens. Ovary in variable position relative to testes: pre-ovarian distance is greater than the pre-testicular distance in the specimen from *C. urodeta*, in 13 of 16 from *E. areolatus*, 8 of 13 from *E. cyanopodus* and 10 of 22 from *E. maculatus*.

**Table 5.** Measurements and ratios of *Prosorhynchus longisaccatus* from *Epinephelus* spp. % refers to % of body-length.

Host	*Epinephelus areolatus*			*Epinephelus cyanopodus*			*Epinephelus maculatus*		
*n*	16			13			22		
	min	max	mean	min	max	mean	min	max	mean
Length	639	1,203	887	920	1,403	1,134	784	1,160	937
Width	185	382	276	298	471	359	191	386	297
Previtelline distance	142	239	176	167	293	212	105	197	167
Precaecal distance	194	347	255	263	420	342	225	348	266
Pre-uterine distance	177	348	260	267	441	322	184	339	285
Pre-mouth distance	359	499	433	502	621	567	367	497	449
Pretesticular distance	250	464	339	334	548	441	272	402	339
Pre-ovarian distance	299	467	367	308	590	460	295	416	338
Rhynchus length	146	262	192	209	347	263	159	244	204
Rhynchus width	135	300	182	178	307	245	131	223	170
Rhynchus to vitellarium distance	0	26	4	0	0	0	0	1	0
Rhynchus to uterus distance	0	148	64	0	148	63	0	291	91
Rhynchus to caecum distance	0	148	62	35	129	81	2	341	81
Long vitelline field	147	337	218	242	355	282	131	292	215
Short vitelline field	99	280	192	146	309	236	108	283	194
Caecum length	101	164	131	94	177	142	88	154	121
Caecum width	65	134	100	77	157	104	66	133	94
Pharynx length	44	72	56	56	86	72	49	78	62
Pharynx width	49	78	58	58	81	75	46	88	65
Ovary length	54	112	77	66	122	99	54	102	76
Ovary width	34	124	69	65	111	92	41	109	68
Distance between ovary and anterior testis	0	66	6	0	0	0	0	16	1
Anterior testis length	59	147	92	85	149	118	58	117	86
Anterior testis width	51	133	82	78	121	97	42	117	75
Distance between testes	0	95	24	0	107	38	0	179	43
Posterior testis length	58	141	92	84	156	113	50	118	85
Posterior testis width	48	149	87	73	135	114	42	106	76
Posterior testis to cirrus-sac	0	23	1	0	0	0	0	59	5
Cirrus-sac length	206	439	284	294	483	377	240	461	331
Cirrus-sac width	84	178	108	112	188	138	82	165	127
Seminal vesicle length	83	150	117	111	211	161	72	155	115
Seminal vesicle width	27	54	40	40	92	66	21	74	42
Pars prostatica length	243	655	348	401	401	401	275	766	424
Pars prostatica width	43	83	63	61	81	69	28	91	57
Post-testicular distance	250	596	390	410	710	518	350	583	436
Post-vitelline distance	341	716	504	513	848	659	435	682	556
Cirrus-sac reach	347	668	455	496	747	587	435	653	518
Post-ovarian distance	269	690	442	484	744	574	408	705	521
Post-genital pore distance	16	115	67	59	118	86	36	98	75
Egg length	26	36	30	24	33	27	24	40	33
Egg width	17	21	18	14	23	18	17	25	21
Width %	20.9	41.5	31.6	25.0	43.1	32.0	23.0	45.8	31.6
Previtelline distance %	14.2	25.4	20.2	14.7	23.8	18.7	11.4	21.6	17.9
Precaecal distance %	23.6	34.1	29.0	26.2	34.1	30.1	23.9	37.8	28.5
Pre-uterine distance %	19.4	38.2	29.4	21.8	40.1	28.6	20.0	36.9	30.5
Pre-mouth distance %	39.9	56.1	49.6	44.0	54.7	49.4	41.4	58.2	47.7
Pretesticular distance %	27.3	47.3	38.9	30.5	46.3	39.0	29.6	44.4	36.4
Pre-ovarian distance %	32.6	50.7	42.3	33.5	48.0	40.5	31.7	46.5	36.2
Rhynchus length %	17.8	24.8	21.9	18.4	26.5	23.2	17.4	26.8	21.9
Rhynchus width % rhynchus length	70.3	114	94.3	71.8	119	94.5	60.4	103.1	83.6
Longest vitelline field %	16.7	28.3	24.6	19.6	32.2	25.1	16.1	29.1	22.9
Caecal length %	9.81	21.8	15.3	9.44	14.8	12.5	10.2	15.4	12.9
Ovary length %	6.12	11.6	8.79	6.02	11.1	8.73	6.46	11.5	8.11
Anterior testis length %	7.28	16.0	10.3	7.65	12.1	10.4	6.83	12.1	9.11
Distance between testes %	0	8.63	2.50	0	10.8	3.42	0	19.4	4.53
Posterior testis %	7.37	15.4	10.2	7.62	12.2	9.90	5.67	11.6	8.98
Posterior testis to cirrus-sac %	0	2.28	0.14	0	0	0	0	6.50	0.49
Cirrus-sac length %	25.4	36.9	32.2	26.5	43.0	33.2	26.2	43.6	35.4
Seminal vesicle length % of cirrus-sac length	38.8	56.2	44.8	37.8	43.7	40.7	20.5	46.5	35.4
Post-testicular distance %	37.7	55.1	43.7	36.9	53.1	45.8	40.1	54.3	47.3
Post-vitelline distance %	50.9	66.3	56.4	50.7	64.0	57.9	52.2	64.5	59.4
Cirrus-sac reach %	39.6	61.7	51.9	45.1	59.6	51.7	43.8	63.7	55.5
Post-ovarian distance %	40.8	58.6	48.9	45.7	56.7	50.6	46.7	61.2	55.4
Post-genital pore distance %	2.42	10.6	7.65	5.62	10.5	7.66	4.29	10.5	7.96

#### Discussion

Our study of this species is based on 52 measured specimens. In our visual key only four species showed no non-overlapping features with our specimens, namely *P. atlanticus, P. longisaccatus*, *P. epinepheli* and *P. lafii* (Table 6). We consider that our specimens conform to the species *P. longisaccatus*, a species originally reported from a “leche”, a serranid from off New Caledonia [9]. Later, we [5] considered our specimens from *E. cyanopodus* as this species and then [19] reported *E. areolatus*, and *E. maculatus* as hosts; all these reports are from New Caledonia. In the latter paper we reported the specimen from *C. urodeta* as *Prosorhynchus* sp.

**Table 6.** Comparisons of *Prosorhynchus longisaccatus*, green shading shows minor distinctions.

	Length	Width %	Rhynchus L %	Previtellarium %	Pre-Uterine %	Pre-mouth %	Post-testicular %	Cirrus-sac reach %	Eggs	Reference
*P. longisaccatus* ex C. *urodeta*	863	40	27	23	35	55	45	51	30 × 20	new data
*P. longisaccatus* ex *E. areolatus*	639-1,203	21–41	18–25	14–25	19–38	40–56	38–55	40–62	26–36 × 17–21	new data
*P. longisaccatus* ex *E. cyanopodus*	920–1,403	25–43	18–27	15–24	22–40	44–55	37–53	45–60	24–33 × 14–23	new data
*P. longisaccatus* ex *E. maculatus*	784–1,160	23–46	17–27	11–22	20–37	41–58	40–54	44–64	24–40 × 17–25	new data
*P. longisaccatus* Durio & Manter, 1968	1,096–1,201	29–30	24	21	33	52	43	48	30–33 × 17–32	[9]
*P. longisaccatus* Durio & Manter, 1968	**1,888–2,088**	29–40	**14–17**	14	39	53	**25**	**27**	30–32 × 16–23	[42]
*P. atlanticus* Manter, 1940	705–1,677	18–28	17–24	22–27	**41–52**	45–47	39–45	**36–39**	27–34 × 14–22	[24]
*P. atlanticus* Manter, 1940	996–1,047	27–33	24–26	23–25	**36–48**	45–48	38–44	**36–42**	31–36 × 18–20	[5]
*P. epinepheli* Yamaguti, 1939	1,250–2,350	40–43	14–19	14	22	54	41	41	28–30 × 18–21	[51]
*P. lafii* Bott & Cribb, 2009	1,040–1,184	**15–22**	18	26	34	44	49	47	29–30 × 15–16	[2]

*Prosorhynchus atlanticus* Manter, 1940 is an Atlantic species, originally described in the serranids, the black grouper *Mycteroperca bonaci* (Poey), the gag *Mycteroperca microlepis* (Goode & Bean) and the yellowfin grouper *Mycteroperca venenosa* (Linnaeus) off Florida [25]. The ovary is, apparently, always pre-testicular, the uterus almost never reaches anteriorly to ovary (only slightly in 1 of 29) and the cirrus-sac reach is generally smaller (Table 6).

*Prosorhynchus epinepheli* Yamaguti, 1939 was originally described from the Hong Kong grouper *Epinephelus akaara* (Temminck & Schlegel) (Serranidae) from the Inland Sea of Japan [52]. The name has been widely used subsequently for *Prosorhynchus* specimens from serranids [8], although some may be misidentified. *P. longisaccatus* is closely similar to *P. epinepheli*. We believe that either *P. epinepheli* or *P. longisaccatus* is the most appropriate identification, particularly as the variable position of the ovary, which is anterior to (and overlapping) the testes or between the testes in our specimens is similar to that described for both of these species. Yamaguti [52] described the position of the ovary in *P. epinepheli* as “overlapping right testis or entirely on its dorsal side (in the type it lies anterodorsal to the right testis, but may be dorsal, dorsolateral or posterodorsal to it)”. Durio & Manter [9] found that in *P. longisaccatus* the ovary is “to the right of, or partly posterior to, anterior testis”. This sheds some doubt on the generic classification of the worm, the variation of which includes a characteristic feature of the genus *Neidhartia* Nagaty, 1937, which according to Overstreet & Curran [33] is “Ovary at level between testes”. Durio & Manter [9] compared their new species to *P. epinepheli,* using Yamaguti’s [52] original description and new material reported from the honeycomb grouper *Epinephelus merra* Bloch, 1793 off Heron Island, southern Great Barrier Reef. It should be noted, however, that Bott & Cribb [2] examined one of Durio & Manter’s “*P. epinepheli*” specimens and considered that it belonged to their new species *P. jexi*, and that the host was most probably not *E. merra*, but the similar species, the longfin grouper *Epinephelus quoyanus* (Valenciennes), which is much commoner in the waters around Heron Island (see also [20]). Durio & Manter [9] summarised the differences between *P. epinepheli* and *P. longisaccatus* as “(1) the uterus does not extend even to midatrial level, whereas in all specimens of *P. epinepheli* it extends postatrially; (2) the rhynchus is wider, and the arrangement of muscles at its anterior edge gives a distinctive appearance”. The first distinction probably relies just on the amount of eggs produced and the second is rather vague and difficult to assess. It seems quite possible that these species are synonymous. There appear to be no morphological criteria for separating these species and we are recognising this species based on the locality of collection, and expect the status of this worm to be elucidated or at least clarified by molecular studies at present in progress.

*Prosorhynchus lafii* Bott & Cribb, 2009 from the brown-marbled grouper *Epinephelus fuscoguttatus* (Forsskål) from off Heron Island, Great Barrier Reef [2] differs from *P. longisaccatus* in the vitelline fields which are “tight lateral clusters at level of caecum”. It is probably a more slender worm than *P. longisaccatus* (Table 6). The ovary is anterior to, but overlapping, the anterior testis.

Suriano & Martorelli [45] reported *P. longisaccatus* in the Remo flounder *Oncopterus darwinii* Steindachner (Pleuronectidae) off Buenos Aires Province, Argentina. It is larger than previously described for this species, with a shorter post-testicular region and cirrus-sac reach, and probably a shorter rhynchus (Table 6). In agreement with Etchegoin et al. [12] we believe that these worms are not conspecific with the worms from serranids in the Pacific Ocean.

### *Prosorhynchus serrani* Durio & Manter, 1968 (Figures 11, 12)

(syn. *Prosorhynchus crucibulus* (Rudolphi, 1819) from *Serranus louti* of Nagaty (1937))

urn:lsid:zoobank.org:act:1B73DB12-40AC-419C-9986-EE6EB612A6AF

Hosts: *Variola albimarginata* Baissac, Serranidae, white-edged lyretail; *Variola louti* (Forsskål), Serranidae, yellow-edged lyretail.

Site: digestive tract.

Locality: *V. albimarginata*, Off Ever Prosperity, external slope, depth 60m (22°27′S, 166°21′E, 07/11/2006); *V. louti*, Near Passe de Dumbéa (22°20′00″S, 166°15′00″E, 01/03/2006, 02/03/2006); Off Ever Prosperity, external slope, depth 60m (22°27′S, 166°21′E, 07/11/2006); Récif Kué, External slope (22°34′892S, 166°29′673E,21/06/2007); External Slope of Récif Toombo (22°33′866S, 166°26′597E, 09/10/2007); Récif Toombo (22°33′172S, 166°26′589E, 20/11/2007).

Prevalence: *V. albimarginata,* 1 of 1; *V. louti*, 6 of 10 (60%).

Vouchers: *V. albimarginata*, MNHN JNC2115; *V. louti*, JNC1756, JNC1757, JNC2117, JNC2198, JNC2301, JNC2401; BMNH 2007.11.14.44, 2013.11.18.13-14.

#### Description

It should be noted that the uterus reaches anteriorly beyond the ovary. Measurements and ratios are given in Table 7.

**Table 7.** Measurements and ratios of *Prosorhynchus serrani*. % refers to % of body-length.

Host	*Variola albimarginata*	*Variola louti*		
*n*	1	10		
		min.	max.	mean
Length	1,322	1,163	2,321	1,775
Width	253	169	311	224
Previtelline distance	314	352	714	524
Precaecal distance	395	409	963	694
Pre-uterine distance	379	443	919	670
Pre-mouth distance	630	611	1,263	985
Pretesticular distance	628	615	1,396	1,046
Pre-ovarian distance	582	571	1,301	976
Rhynchus length	177	147	247	187
Rhynchus width	141	102	139	122
Rhynchus to vitellarium distance	136	158	505	337
Rhynchus to uterus distance	377	253	871	514
Rhynchus to caecum distance	218	214	875	579
Long vitelline field	350	193	637	448
Short vitelline field	300	201	547	374
Caecum length	177	0	327	200
Caecum width	101	60	121	84
Pharynx length	0	0	78	53
Pharynx width	0	0	76	54
Ovary length	107	77	134	108
Ovary width	81	63	128	94
Distance between ovary and anterior testis	0	0	0	0
Anterior testis length	126	84	178	126
Anterior testis width	108	76	162	116
Distance between testes	65	13	115	67
Posterior testis length	138	72	168	128
Posterior testis width	109	70	138	111
Posterior testis to cirrus-sac	0	0	0	0
Cirrus-sac length	338	222	359	294
Cirrus-sac width	80	77	135	103
Seminal vesicle length	0	0	130	43
Seminal vesicle width	0	0	69	17
Pars prostatica length	0	0	387	137
Pars prostatica width	0	0	87	51
Post-testicular distance	371	263	577	404
Post-vitelline distance	658	508	1,041	773
Cirrus-sac reach	444	376	579	491
Post-ovarian distance	635	493	908	676
Post-genital pore distance	112	0	203	91
Egg length	24	24	33	28
Egg width	13	15	22	18
Width %	19	9.9	17.5	13.0
Previtelline distance %	24	26.3	33.3	29.4
Precaecal distance %	30	31.8	43.4	38.7
Pre-uterine distance %	29	26.2	52.1	38.4
Pre-mouth distance %	48	52.1	59.3	55.5
Pretesticular distance %	48	49.4	65.2	58.5
Pre-ovarian distance %	44	45.4	60.7	54.6
Rhynchus length %	13	7.8	16.7	11.0
Rhynchus width % rhynchus length	80	55.1	79.2	66.2
Longest vitelline field %	27	16.6	29.7	24.9
Caecal length %	13	0	15.7	11.2
Ovary length %	8	4.74	9.02	6.23
Anterior testis length %	10	5.49	9.73	7.21
Distance between testes %	5	1.12	5.84	3.71
Posterior testis %	10	4.77	12.24	7.39
Posterior testis to cirrus-sac %	0	0	0	0
Cirrus-sac length %	26	13.7	22.1	17.1
Seminal vesicle length % of cirrus-sac length	0	0	40.0	14.8
Post-testicular distance %	28	17.6	31.7	23.0
Post-vitelline distance %	50	37.3	50.3	43.8
Cirrus-sac reach %	34	23.7	39.3	28.6
Post-ovarian distance %	48	33.2	47.7	38.5
Post-genital pore distance %	8	0	8.75	4.96

#### Discussion

*Prosorhynchus serrani* is known previously only from the yellow-edged lyretail *Variola louti* (Forsskål) (Serranidae) from the Red Sea and off New Caledonia [9, 29]. Our specimens appear indistinguishable from those described by these authors.

This species is very similar to several other species and their relationships will probably only be resolved by molecular means. However, there seem to be minor morphological features which may allow the continued recognition of the *Variola* parasites as distinct (Table 8). Of those with no distinction in the parameters used in the visual key two can immediately be distinguished by other features.

**Table 8.** Comparisons of *Prosorhynchus serrani*, green shading shows minor distinctions.

	Length	Width %	Rhynchus L %	Previtelline %	Pre-Uterine %	Pre-mouth %	Post-testicular %	Cirrus-sac reach %	Eggs	Reference
*P. serrani* ex *V. albimarginata*	1,322	19	13	24	29	48	28	34	24 × 13	new data
*P.serrani* ex *V. louti*	1,163–2,321	10–18	8–17	26–33	26–52	52–59	18–32	24–39	24–33 × 15–22	new data
*P. serrani* Durio & Manter, 1968	1,027–2,245	22	12–18	20	17	49	27	33	24–29 × 15–21	[9]
*P. serrani* Durio & Manter, 1968	816–1,826	30–32	15–16	30–34	31	53–62	24–31	31–36	25–29 × 17–21	[27]
*P. attenuatus* Siddiqi & Cable, 1960	693–1,107	17–20	9–13	28	27	55	30	33	19–21 × 13–15	[41]
*P. caballeroi* Gupta & Ahmad, 1976	**2,980**	14	**6**	**42**	36	57	18	25	19–21 × 11–13	[17]
*P. caudovatus* Manter, 1940	2,000–4,000	17–20	10–17	18–31	25–46	39–44	**38**–**46**	29–35	**38**–**45** × **19**–**22**	[10]
*P. caudovatus* Manter, 1940	1,715–2,245	27–30							**32**–**43** × **21**–**25**	[14]
*P. caudovatus* Manter, 1940	3,672	12	11	21	37	38	**50**	36		[4]
*P. conorjonesi* Bott & Cribb 2009	**1,904**–**3,360**	**6**–**7**	9–12	28	52	**45**	**31**	**20**	31–32 × 16	[2]
*P. jexi* Bott & Cribb, 2009	1,104–1,424	19–25	15	20	32	**43**	**41**	37	32–33 × 16	[2]
*P. milleri* Bott & Cribb, 2009	1,392–1,648	13–14	9	31	51	56	24	25	25 × 16	[2]
*P. robertsthomsoni* Bott & Cribb, 2009	1,072–1,408	18–23	10–11	27	24	48	31	35	29–30 × 16	[2]
*P. thapari* Manter, 1953	1,778–2,282	15–16	12	33	44	53	25	28	27–34 × 19–22	[25]
*P. truncatus* Verma, 1936	1,760–2,600	23–24	16–20	30	?	**63**	21	**20**	35–40 × 18–20	[46]

*P. attenuatus* Siddiqi & Cable, 1960 from the Atlantic bumper *Chloroscombrus chrysurus* (Girard) (Carangidae) off Puerto Rico was described with a “spherical, suckerlike” rhynchus and it certainly looks like a sucker in the illustration. The pars prostatica is described as “tubular” and appears straight in the illustration [43], thus indicating that it may have been placed in the wrong subfamily.

*P. caudovatus* Manter, 1940 (syn. *P. crucibulus* of Eckmann (1932)) from serranids in the waters around Africa [4, 10, 13, 14, 46] has distinctive filamented eggs.

Other similar species are:

*Prosorhynchus caballeroi* Gupta & Ahmad, 1976 known from one specimen from the shrimp scad *Alepes djedaba* (Forsskål) (as *Caranx kalla* Cuvier) (Carangidae) in the Bay of Bengal [17] grows larger than *P. serrani*, with a smaller rhynchus and a longer previtelline distance.

*Prosorhynchus conorjonesi* Bott & Cribb 2009 from the barramundi cod *Cromileptes altivelis* (Valenciennes) (Serranidae) on the Great Barrier Reef [2] grows larger than *P. serrani*, is much narrower, with a more anterior mouth and a shorter cirrus-sac reach.

*Prosorhynchus jexi* has a more anterior mouth than *P. serrani* and a longer post-testicular region [2, 9].

*Prosorhynchus milleri* Bott & Cribb, 2009 based on two specimens from *Variola louti* from Lizard Island, Great Barrier Reef [2] is very similar to *P. serrani* and from one of the same host species. It is said to differ from *P. serrani* in that the latter has “a uterus that extends anterior to the vitelline follicles into the anterior quarter of the body”. Our results complicate things in that the anterior uterine extent varies considerably in our specimens from *V. louti*. Judging from the illustration of *P. milleri* in Bott & Cribb [2] the pre-uterine extent is about 51% of body-length and judging from Durio & Manter’s [9] illustration of *P. serrani* this ratio is about 17%. Durio & Manter [9] considered *P. crucibulum* from *V. louti* of Nagaty [29] a synonym of *P. serrani* and judging from Nagaty’s illustration the pre-uterine distance is about 31% of body-length. This ratio in our worms varies between 26 and 52%, and without a distinct bimodal pattern (26, 29, 30, 32, 33, 38, 39, 40, 45, 49 and 52%). It may well be that there are two forms here, but we do not as yet have enough data to be certain where to draw the line.

*Prosorhynchus robertsthomsoni* Bott & Cribb, 2009 is very similar to *P. serrani* but apparently has a distinctly different shaped rhynchus, in that it has a blunt rounded posterior extension in contrast to the distinct narrow elongate posterior extension of the *P. serrani* rhynchus [2]. It may be that the vitellarium reaches slightly more posterior in *P. serrani* in that the follicles extend just posterior to the pharynx, rather than just to the pharynx.

*Prosorhynchus thapari* Manter, 1953 was based on 17 specimens from the spotted coralgrouper *Plectropomus maculatus* (Bloch) (Serranidae) from off Fiji [26]. We can detect no morphological distinctions from *P. serrani* and retain the species as separate based on host distinction, and the knowledge that as yet unpublished studies indicate some specificity and cryptic speciation in the genus. Nevertheless, it may well be that this is the oldest valid name for this species.

*Prosorhynchus truncatus* Verma, 1936 is based on two specimens, one ovigerous and lost and the other without eggs, from the intestine of the river catfish *Cephalocassis jatia* (Hamilton) (as *Arius j.*) (Ariidae) off Puri, Bay of Bengal [49]. It has a more posteriorly situated mouth and a shorter cirrus-sac reach.

### *Prosorhynchus freitasi* Nagaty, 1937 (Figures 13, 14)

urn:lsid:zoobank.org:act:D5FF3B1F-10B1-4447-ACB7-C7D544C36AFE

**Figure 13–18.**
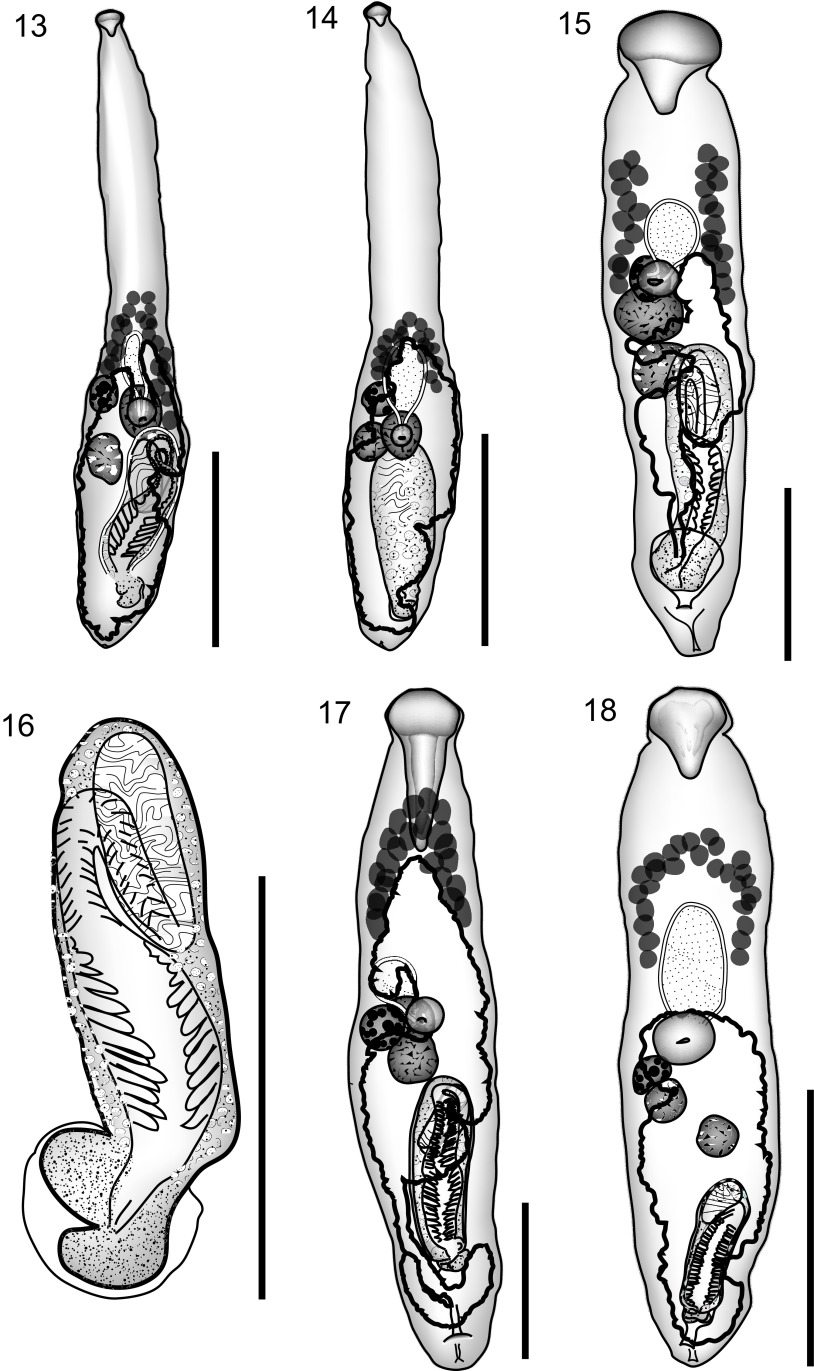
13: *Prosorhynchus freitasi* Nagaty, 1937 from *Plectropomus leopardus*, uterus in outline. 14: *Prosorhynchus freitasi* Nagaty, 1937 from *Plectropomus laevis*, uterus in outline. 15: *Prosorhynchus luzonicus* Velasquez, 1959, uterus in outline. 16: *Prosorhynchus luzonicus* Velasquez, 1959, cirrus-sac. 17: *Prosorhynchus* sp. A ex *Epinephelus morrhua.* Ventral view, uterus in outline. 18: *Prosorhynchus* sp. B ex *Epinephelus coioides.* Ventral view, uterus in outline. Scale bars: 500 μm (Figs. 13–15, 17, 18); 200 μm (Fig. 16).

Host: *Plectropomus laevis* (Lacepède), Serranidae, blacksaddled coralgrouper; *Plectropomus leopardus* (Lacepède), Serranidae, leopard coralgrouper.

Site: digestive tract.

Localities: *P. laevis* Off Ouano (21°49′430S, 166°44′278E, 25/10/2007); *P. leopardus* Grande Rade, Nouméa (22°15′S 166°24E, 23/10/2007), Off Ouano (21°49′430S, 166°44′278E, 25/10/2007), Between Larégnière and Récif Crouy (22°20′702S, 166°19′295E, 05/05/2008).

Prevalence: *P. laevis* 1 of 2 (50%); *P. leopardus* 5 of 7 (71%).

Vouchers: *P. laevis* JNC2339; *P. leopardus* MNHN JNC2333A, JNC 2334, JNC2340, JNC2513, JNC 2514; BMNH 2013.11.18.7-12.

#### Discussion

Table 9 measurements, Table 10 comparisons.

**Table 9.** Measurements and ratios of *Prosorhynchus freitasi* and *P. luzonicus*. % refers to % of body-length.

Species	*Prosorhynchus freitasi*			*Prosorhynchus freitasi*			*Prosorhynchus luzonicus*		
Host	*Plectropomus leopardus*			*Plectropomus laevis*			*Epinephelus coioides*		
*n*	17			6			14		
	min.	max.	mean	min.	max.	mean	min.	max.	mean
Length	848	1,650	1,239	1,365	1,591	1,502	792	1,172	925
Width	134	363	207	214	290	255	129	288	202
Previtelline distance	322	778	560	633	860	717	142	221	171
Precaecal distance	358	923	640	736	878	795	219	311	257
Pre-uterine distance	343	918	629	693	928	807	263	409	336
Pre-mouth distance	573	1,134	888	947	1,131	1,020	327	471	381
Pretesticular distance	488	1,085	771	837	1,095	959	315	456	372
Pre-ovarian distance	452	1,052	729	801	1,039	912	276	407	338
Rhynchus length	37	61	51	55	63	60	126	170	142
Rhynchus width	42	67	53	48	63	54	99	170	130
Rhynchus to vitellarium distance	281	717	510	564	802	658	0	72	29
Rhynchus to uterus distance	296	874	587	646	917	754	133	272	194
Rhynchus to caecum distance	314	900	602	665	876	752	78	163	114
Long vitelline field	119	329	205	194	270	235	186	344	251
Short vitelline field	112	249	170	153	223	182	154	257	207
Caecum length	86	190	128	148	180	161	80	125	105
Caecum width	22	87	51	51	67	59	58	108	79
Pharynx length	36	71	51	47	57	52	42	66	53
Pharynx width	41	73	52	56	62	59	46	75	58
Ovary length	51	105	79	87	108	95	58	95	74
Ovary width	37	97	66	73	96	82	54	79	65
Distance between ovary and anterior testis	0	38	9	0	47	8	0	0	0
Anterior testis length	63	140	95	92	138	114	69	99	82
Anterior testis width	44	126	80	74	114	93	66	111	80
Distance between testes	0	53	13	0	18	3	0	39	10
Posterior testis length	57	141	97	89	136	114	69	92	80
Posterior testis width	47	113	73	80	104	91	71	92	80
Posterior testis to cirrus-sac	0	0	0	0	0	0	0	0	0
Cirrus-sac length	204	380	281	303	360	330	230	360	281
Cirrus-sac width	57	120	89	89	157	115	70	124	95
Seminal vesicle length	64	197	139	?	?	?	101	165	138
Seminal vesicle width	17	61	48	?	?	?	37	63	51
Pars prostatica length	360	509	444	?	?	?	302	439	357
Pars prostatica width	55	101	73	?	?	?	54	97	73
Post-testicular distance	163	394	284	283	454	344	308	534	383
Post-vitelline distance	331	629	473	493	605	536	411	636	506
Cirrus-sac reach	266	529	398	455	496	465	363	532	426
Post-ovarian distance	308	552	427	455	558	485	410	682	510
Post-genital pore distance	24	71	57	39	82	68	82	160	108
Egg length	24	32	27	24	28	26	27	35	30
Egg width	13	21	17	16	19	17	15	20	18
Width %	12.2	22.8	16.6	13.5	20.0	17.0	16.3	25.2	21.7
Previtelline distance %	35.3	50.0	44.8	43.9	54.2	47.6	15.6	20.8	18.5
Precaecal distance %	39.3	56.5	51.1	51.1	55.6	53.5	25.2	30.6	27.6
Pre-uterine distance %	37.7	60.0	50.1	50.2	58.8	53.6	31.0	46.6	36.7
Pre-mouth distance %	63.2	71.2	66.6	66.7	71.6	68.6	37.7	44.3	41.3
Pretesticular distance %	53.6	68.1	61.9	61.3	69.0	63.7	37.9	43.2	40.3
Pre-ovarian distance %	49.6	66.0	58.3	57.3	65.5	60.6	33.0	39.8	36.6
Rhynchus length %	3.2	5.0	4.2	3.6	4.4	4.0	12.9	17.6	15.5
Rhynchus width % rhynchus length	83.0	123.8	104.9	75.6	102.1	89.2	76.3	105.5	91.4
Longest vitelline field %	13.4	20.6	16.5	12.2	17.9	15.7	23.5	29.6	27.1
Caecal length %	8.0	13.9	10.4	9.7	11.3	10.9	9.4	14.0	11.3
Ovary length %	5.24	8.8	6.47	5.47	7.5	6.33	7.10	8.8	7.98
Anterior testis length %	5.04	10.1	7.7	5.82	9.6	7.6	6.55	10.7	8.9
Distance between testes %	0	3.33	0.98	0	1.11	0.19	0	3.73	0.97
Posterior testis length %	4.6	10.8	8.0	6.0	8.7	7.6	6.9	10.2	8.7
Posterior testis to cirrus-sac %	0	0	0	0	0	0	0	0	0
Cirrus-sac length %	17.3	25.2	22.0	21.0	24.3	22.3	25.4	36.9	30.5
Seminal vesicle length % of cirrus-sac length	30.1	55.7	43.6	?	?	?	37.7	61.4	48.8
Post-testicular distance %	18.7	29.3	23.1	17.8	28.5	23.0	37.8	45.6	41.3
Post-vitelline distance %	32.2	45.1	38.5	31.1	38.2	35.7	50.5	59.3	54.7
Cirrus-sac reach %	26.6	35.1	31.2	29.0	33.7	31.4	42.6	50.9	46.3
Post-ovarian distance %	28.9	44.0	34.9	28.7	35.0	32.4	51.8	58.5	55.1
Post-genital pore distance %	2.12	6.29	4.17	2.89	5.65	4.60	10.24	14.96	11.72

**Table 10.** Comparisons of *Prosorhynchus freitasi*, blue shading shows major distinctions, green shading minor distinctions.

	length	width %	Rhynchus L %	Previtelline %	Pre-Uterine %	Pre-mouth %	Post-testicular %	Cirrus-sac reach %	Eggs	Reference
*P. freitasi* ex *Plectropomus leopardus*	848–1,506	12–20	4–5	35–47	38–55	63–68	19–29	28–35	24–30 × 13–21	new data
*P. freitasi* ex *Plectropomus laevis*	1,365–1,591	14–20	4	44–54	50–59	67–72	18–24	29–34	24–28 × 16–19	new data
*P. freitasi* Nagaty, 1937	919–1,870	10–23	4–6	49–50	61	63–65	22–26	27–28	21–29 × 17–21	[27]
*P. freitasi* Nagaty, 1937	1,216–1,564	12–17	4–5	39	46	60	29	?	24–26 × 14–15	[3]
*P. arabiana* Srivastava, 1938	**3,300**–**4,500**	12–13	6–9	**64**	41	62	18	**22**	23 × 12	[43]
*P. heronensis* Bott, Miller & Cribb, 2013	1,040–1,104	15–19	**8**	45	55	70	17	?	26–27 × 13–15	[3]
*P. indicus* Madhavi, 1974	**3,360**–**4,480**	11–12	4–5	50	37	60	16	**19**	**17**–**19** × **8**–**11**	[23]
*P. lesteri* Bott, Miller & Cribb, 2013	1,341–2,320	13–18	**10**–**11**	41	51	60	23	30	19–26 × 14–15	[3]
*P. milleri* Bott & Cribb, 2009	1,392–1,648	13–14	**9**	31	51	**56**	24	25	25 × 16	[2]
*P. munozae* Bott, Miller & Cribb, 2013	700–1,040	17–18	4–6	46	57	68	24	28	**31–36 × 19–23**	[3]
*P. orientalis* Gupta & Ahmad, 1976	**3,600**	**8**	5	47	47	65	**14**	**14**	22–30 × 11–17	[17]
*P. plectropomi* Bott, Miller & Cribb, 2013	1,024–1,280	13–16	4–5	47	**44**	66	24	29	24–26 × 14–15	[3]
*P. stunkardi* Siddiqi & Cable, 1960	1,056–1,227	16–22	5–10	38	58	**56**	20	24	**16**–**18** × **11**–**15**	[41]
*P. thapari* Manter, 1953	1,778–2,282	15–16	**12**	33	44	**53**	25	28	27–34 × 19–22	[25]
*P. truncatus* Verma, 1936	1,760–2,600	23–24	**16**–**20**	30	?	63	21	20	**35**–**40** × **18**–**20**	[46]
*P. wrightae* Bott, Miller & Cribb, 2013	800–1,088	16–21	6–7	38	**30**	66	22	33	20–24 × 12–13	[3]

In terms of the parameters used in the visual key there are no differences between our specimens from *Plectropomus laevis* and *Prosorhynchus freitasi* as described from “*Serranus guttatus*” from the Red Sea [29]. According to Froese & Pauly [15] *S. guttatus* is now known as the peacock hind *Cephalopholis argus* (Bloch) (Serranidae). It has also been reported in *Epinephelus* sp. and the spotted coralgrouper *Plectropomus maculatus* (Bloch) (Serranidae) from off New Caledonia [9] and *Plectropomus leopardus* and *Plectropomus laevis* from the Great Barrier Reef [3]. It has an unusual morphology in that all the internal organs are restricted to the posterior half of the body and the rhynchus is relatively small.

Bott et al. [3] described six *Prosorhynchus* species from *Plectropomus* spp. on the Great Barrier Reef, five of which are new and one, *P. freitasi* already known. They are mostly distinguished by minor morphological characters and by analysis of ITS2 rDNA sequences. *P. lesteri* is distinguished by its distinctly larger rhynchus. *P. wrightae* differs in the pre-uterine extent, being the only one of these species where the uterus extends well beyond the vitellarium anteriorly. *P. heronensis* also has a larger rhynchus, although not as large as in *P. lesteri,* and a distinct U-shaped seminal vesicle. In *P. plectropomi* the uterus extends to, or just anterior to the anterior extent of the vitellarium, apparently forcing the anterior follicles apart, breaking up the continuous arc found in other related species. *P. munozae* is a rather small worm, but with larger eggs. Our specimens agree closely with Bott et al.’s [3] description of *P. freitasi*.

### *Prosorhynchus luzonicus* Velasquez, 1959 (Figures 15, 16)

urn:lsid:zoobank.org:act:25F350A1-F852-4CA9-91D8-A288F9B3F7DD

Host: *Epinephelus coioides* (Hamilton) (Serranidae), orange-spotted grouper.

Site: Digestive tract.

Locality: Fish Market, Nouméa (14/10/2010).

Prevalence: 1 of 1.

Vouchers: MNHN JNC3277; BMNH 2013.11.18.24.

#### Discussion

See Table 9 for measurements and Table 11 for morphological comparisons. These specimens from *E. coioides* are clearly different from those from this host mentioned below as *Prosorhynchus* sp. B, particularly in pre-mouth distance (see Table 13) and vitelline distribution, but also in post-testicular distance and cirrus-sac reach. On the other hand they are very similar to *P. luzonicus* as originally described [48] from the barramundi *Lates calcarifer* (Bloch), (Latidae) from Malabon, Rizal, Luzon island, Philippines. It has been reported in *E. coioides* in Lampung Bay, southern Sumatra, Indonesia [34–36]. Rückert [35] described and illustrated this species from *Epinephelus fuscoguttatus*, also from Lampung Bay, and later reported it again in this host, both in culture and in the wild [38]. It is slightly disconcerting that Rückert et al. [37] failed to find this species in *L. calcarifer* in her study of Lampung Bay. Two useful, but not infallible, recognition features are the separated vitelline fields (occasionally they appear to form an arch), and the mainly postovarian uterus (but according to the figure and illustration by Rückert [35] this is not invariable). Our specimens differ slightly from Velasquez’s [48] description in the greater extent of the cirrus-sac reach as a proportion of body-length (43–51% vs. about 38%). Rückert [35] shows a proportion of about 39%.

**Table 11.** Comparisons of *Prosorhynchus luzonicus*, green shading shows minor distinctions.

	Length	Width %	Rhynchus L %	Previtelline %	Pre-Uterine %	Pre-mouth %	Post-testicular %	Cirrus-sac reach %	Eggs	Reference
*P. luzonicus* Velasquez, 1959	792–1,172	16–25	13–18	16–21	31–47	38–44	38–46	43–51	27–35 × 15–20	new data
*P. luzonicus* Velasquez, 1959	1,060–2,020	22–25	10–19	20	42	39	38	**38**	30–39 × 17–24	[45]
*P. jexi* Bott & Cribb, 2009	1,104–1,424	19–25	15	20	32	43	41	**37**	32–33 × 16	[2]
*P. maternus* Bray & Justine, 2006	**2,052**–**2,227**	19–21	13–17	19–23	34–41	38–40	**45**–**53**	**32**–**39**	27–28 × 14–22	[5]
*P. pacificus* Manter, 1940	1,206–1,444	25–27	14–16	**31**	47	**45**	37	42	**24**–**27** × **12**–**17**	[24]
*P. pacificus* Manter, 1940, types	1,232–1,359	26–30	18–19	**26**–**30**	41–46	**46**–**48**	38–49	44–48	28–31 × 15–16	[5]
*P. robertsthomsoni* Bott & Cribb, 2009	1,072–1,408	18–23	10–11	**27**	**24**	**48**	**31**	**35**	29–30 × 16	[2]
*P. robertsthomsoni* Bott & Cribb, 2009	1,088–1,256	23–24	10–12	18–23	**10**–**26**	**45**–**47**	**31**–**40**	39–48	32–38 × 16–20	new data
*P. squamatus* Odhner, 1905	1,000–1,500	**34**	10–15	**12**	**22**	**49**	**28**	**34**	29–32 × ?	[30]

*Prosorhynchus jexi* is similar, but differs in cirrus-sac extent, in the arched vitelline fields and in the extension of the uterus anterior to the ovary (but note that these latter features appear to be variable in *P. luzonicus*) [2, 9].

*Prosorhynchus maternus* Bray & Justine, 2006 from the Malabar grouper *Epinephelus malabaricus* (Bloch & Schneider) off New Caledonia [5] differs in size, post-testicular region and cirrus-sac reach.

*Prosorhynchus pacificus* Manter, 1940 is an eastern Pacific form, having been reported originally from the serranids, the sailfin grouper *Mycteroperca olfax* (Jenyns), the broomtail grouper *Mycteroperca xenarcha* Jordan and an unidentified grouper off the Galapagos [24]. Later records were summarised by Bray & Justine [5], who re-measured two type-specimens. Slight differences from our specimens can be detected in previtelline, pre-mouth and post-testicular distances, cirrus-sac reach and egg-size range. Some specimens from cultured *E. coioides* in Vietnam have been identified as this species, others as *P. epinepheli* [51].

*Prosorhynchus robertsthomsoni* (see above, including new data) differs slightly in pre-uterus, pre-mouth and post-testicular distances [2].

*Prosorhynchus squamatus* Odhner, 1905 is a Northern Hemisphere species, originally reported from the shorthorn sculpin *Myoxocephalus scorpius* (Linnaeus) (Cottidae) [32], but since reported in many cold-water hosts [16, 21]. It differs from *P. luzonicus* in width, previtelline, pre-uterine, pre-mouth and post-testicular distances, cirrus-sac reach and probably in its arched vitellarium and pre-ovarian uterine extent.

### *Prosorhynchus* sp. A (Figure 17)

*Epinephelus morrhua* (Valenciennes, 1833), Serranidae, comet grouper.

Site: digestive tract.

Locality: Off Récif Kué, deep-sea (22°35′511S, 166°9′893E, 23/01/2008).

Prevalence: 1 of 4 (25%).

Vouchers: MNHN JNC2453; BMNH 2013.11.18.26.

#### Discussion

No species are identical to these two specimens according to the visual key (Tables 12, 13). As only one specimen is in good condition, the worms have not been described as new, but the very elongate rhynchus seems to be a distinguishing feature. Also note that the ovary lies beside the anterior testis.

**Table 12.** Measurements and ratios of *Prosorhynchus* spp. from *Epinephelus* spp. % refers to % of body-length.

Species	*Prosorhynchus* sp. A		*Prosorhynchus* sp. B		
Host	*Epinephelus morrhua*		*Epinephelus coioides*		
*n*	2		3		
			min.	max.	mean
Length	2,157	2,110	1,006	1,297	1,199
Width	470	508	217	294	266
Previtelline distance	310	222	217	263	244
Precaecal distance	?	888	306	414	357
Pre-uterine distance	540	?	451	621	545
Pre-mouth distance	1,035	1,062	570	685	637
Pretesticular distance	964	1,175	556	769	694
Pre-ovarian distance	1,002	1,186	584	729	675
Rhynchus length	513	335	155	175	165
Rhynchus width	231	320	121	155	142
Rhynchus to vitellarium distance	0	malformed	61	95	82
Rhynchus to uterus distance	30	malformed	292	446	379
Rhynchus to caecum distance	?	555	148	237	191
Long vitelline field	454	?	220	262	237
Short vitelline field	478	?	217	275	239
Caecum length	?	275	183	241	217
Caecum width	?	253	113	129	122
Pharynx length	112	126	64	85	78
Pharynx width	104	108	80	114	97
Ovary length	147	146	57	77	64
Ovary width	137	142	50	69	58
Distance between ovary and anterior testis	0	0	0	9	3
Anterior testis length	181	202	70	94	80
Anterior testis width	161	197	75	81	77
Distance between testes	0	0	0	45	15
Posterior testis length	168	171	75	82	78
Posterior testis width	170	169	64	78	73
Posterior testis to cirrus-sac	0	0	0	30	15
Cirrus-sac length	611	593	233	266	251
Cirrus-sac width	198	251	80	120	102
Seminal vesicle length	226	182	?	123	41
Seminal vesicle width	68	74	?	53	18
Pars prostatica length	791	861	?	?	?
Pars prostatica width	142	159	56	84	73
Post-testicular distance	905	569	324	394	368
Post-vitelline distance	1,348	0	566	803	708
Cirrus-sac reach	927	863	355	379	369
Post-ovarian distance	1,001	797	348	519	459
Post-genital pore distance	97	96	42	57	49
Egg length	35	malformed	29	32	30
Egg width	18	malformed	18	21	19
Width %	21.8	24.1	21.6	22.7	22.2
Previtelline distance %	14.4	10.5	19.3	21.6	20.4
Precaecal distance %	?	42.09	27.0	31.9	29.8
Pre-uterine distance %	25.02	?	43.5	47.9	45.4
Pre-mouth distance %	48.0	50.3	50.6	56.7	53.4
Pretesticular distance %	44.7	55.7	55.3	59.3	57.7
Pre-ovarian distance %	46.4	56.2	54.9	58.1	56.4
Rhynchus length %	23.8	15.9	12.6	15.4	13.9
Rhynchus width % rhynchus length	44.9	95.4	78.1	91.0	85.8
Longest vitelline field %	21.1	?	17.6	21.9	19.9
Caecal length %	?	13.0	17.5	18.6	18.1
Ovary length %	6.81	6.92	4.41	5.96	5.36
Anterior testis length %	8.41	9.59	5.99	7.22	6.71
Distance between testes %	0	0	0	3.44	1.15
Posterior testis %	7.79	8.12	5.78	8.13	6.63
Posterior testis to cirrus-sac %	0	0	0	2.34	1.14
Cirrus-sac length %	28.3	28.1	18.0	26.5	21.3
Seminal vesicle length % of cirrus-sac length	37.0	30.8	?	46.3	?
Post-testicular distance %	42.0	27.0	29.7	32.3	30.8
Post-vitelline distance %	62.5	0.0	56.3	61.9	58.8
Cirrus-sac reach %	43.0	40.9	27.4	37.0	31.2
Post-ovarian distance %	46.4	37.8	34.6	40.0	38.0
Post-genital pore distance %	4.48	4.53	3.65	4.39	4.08

One species, *Prosorhynchus epinepheli*, has one major distinguishing feature in the visual key, i.e., width (Table 13). Minor distinguishing features are the pre-mouth distance and the egg-size.

**Table 13.** Comparisons of *Prosorhynchus* spp. from *Epinephelus* spp., blue shading shows major distinctions, green shading minor distinctions.

	Length	Width %	Rhynchus L %	Previtelline %	Pre-Uterine %	Pre-mouth %	Post-testicular %	Cirrus-sac reach %	Eggs	Reference
*P.* sp. in *Epinephelus morrhua*	2,110–2,157	22–24	16–24	11–14	25	48–50	27–42	41–43	35	new data
*P. epinepheli* Yamaguti, 1939	1,250–2,350	**40**–**43**	14–19	14	22	**54**	41	41	**28**–**30** × **18**–**21**	[51]
*P.* sp. in *Epinephelus coioides*	1,006–1,297	22–23	13–15	19–22	44–48	51–57	30–32	27–37	29–32 × 18–21	new data
*P. caudovatus* Manter, 1940	**2,000**–**4,000**	17–20	10–17	18–31	25–46	**39**–**44**	**38**–**46**	29–35	**38**–**45** × **19**–**22**	[10]
*P. caudovatus* Manter, 1940	**1,715**–**2,245**	27–30							**32**–**43** × **21**–**25**	[14]
*P. caudovatus* Manter, 1940	3,672	12	11	21	37	38	50	36		[4]
*P. milleri* Bott & Cribb, 2009	1,392–1,648	**13**–**14**	**9**	31	51	56	**24**	25	**25** × **16**	[2]
*P. pacificus* Manter, 1940	1,206–1,444	25–27	**14**–**16**	**31**	47	**45**	**37**	42	**24**–**27** × **12**–**17**	[23]
*P. pacificus* Manter, 1940, types	1,232–1,359	26–30	**18**–**19**	**26**–**30**	41–46	**46**–**48**	**38**–**49**	**44**–**48**	**28–31 × 15–16**	[5]
*P. paracrucibulus* Velasquez, 1959	1,096–1,600	**30**–**36**	12–16	23	?	57	34	27	none	[45]
*P. truncatus* Verma, 1936	**1,760**–**2,600**	23–24	16–20	**30**	?	**63**	**21**	**20**	**35**–**40** × **18**–**20**	[46]

### *Prosorhynchus* sp. B. (Figure 18)

Host: *Epinephelus coioides* (Hamilton, 1822) (Serranidae), orange-spotted grouper.

Site: Digestive tract.

Locality: Fish Market, Nouméa (27/11/2009).

Prevalence: 1 of 1.

Vouchers: MNHN JNC3140; BMNH 2013.11.18.27.

#### Discussion

Measurements of the three specimens are given in Table 12. Several species are very similar to *Prosorhynchus* sp. B, and show no differences in the visual key but may be distinguished by combinations of minor features (Table 13). More specimens are needed to describe this form as new as so many similar *Prosorhynchus* species are known.

*Prosorhynchus caudovatus* Manter, 1940 (syn. *P. crucibulus* of Eckmann (1932)) from serranids in the waters around Africa [4, 10, 13, 14, 46] has a distinctive filamented egg. It is also distinctly larger than *P.* sp. B, has a more anterior mouth and a longer post-testicular region.

*Prosorhynchus milleri* Bott & Cribb, 2009 based on two specimens from *Variola louti* from Lizard Island, Great Barrier Reef [2] is longer, narrower, with a smaller rhynchus, a longer previtelline region and a shorter post-testicular region. The vitelline fields reach to the pharynx (vs. distinctly anterior).

*Prosorhynchus pacificus* belongs to a group of *Prosorhynchus* spp. with the uterus restricted to the post-ovarian region. In this aspect it differs from *P.* sp. B. It also differs in previtelline distance, pre-mouth distance, post-testicular region and cirrus-sac reach. The vitelline fields reach the ovary (vs. well anterior to the pharynx).

*Prosorhynchus paracrucibulus* Velasquez, 1959 based on three non-ovigerous worms (presumably metacercariae) from the scales (!) of the Buru glass perchlet *Ambassis buruensis* Bleeker (Ambassidae) Manila Bay, Paranaque, Rizal, Luzon Island, Philippines [48]. It is a little wider, with symmetrical testes. The worm is not developed sufficiently enough to recognise, but conceivably it is the metacercaria of a serranid parasite.

*Prosorhynchus truncatus* Verma, 1936 is based on two specimens, one ovigerous and lost and the other without eggs, from the intestine of the river catfish *Cephalocassis jatia* (Hamilton) (as *Arius j.*) (Ariidae) off Puri, Bay of Bengal [49]. It is considerably longer than *P.* sp. It also differs in previtelline distance, pre-mouth distance, post-testicular region and cirrus-sac reach.

*Prosorhynchus* specimens from cultured *E. coioides* in Vietnam have been identified as *Prosorhynchus luzonicus* and *P. epinepheli* [51].

### *Prosorhynchus* sp. immature

Host: *Epinephelus coeruleopunctatus* (Bloch, 1790)

Site: Digestive tract.

Locality: Îlot Lebris, off Ouano (21°50′S, 166°45′E, 25/10/2007).

Prevalence: 1 of 3.

Vouchers: MNHN JNC2338.

#### Discussion

A single unidentifiable immature specimen was recovered from this host species.

## Conclusions

The molecular evidence presented by Bott et al. [3] indicated that there are many distinct, but very similar species of prosorhynchines in serranids, especially *Epinephelus* and *Plectropomus*. The morphological similarity of these forms has led to many problems in identification, and some unlikely combinations of hosts in the literature, as for example the quoted hosts for *Neidhartia neidharti*, which in addition to serranids, includes a belonid and a freshwater siluriform. Recent molecular studies of a wide range of digeneans have indicated that most species exhibit oioxenous or stenoxenous specificity and “that no euryxenous host distribution should be accepted on the basis of morphology only” [28]. Although it is dangerous to identify parasites solely on the basis of their hosts, consideration should be taken of the relatedness of the hosts and the geographical distribution.

Cribb et al. [8] discussed the digenean fauna of epinepheline serranids and found that *Prosorhynchus* was the commonest parasite, both in the Atlantic/Eastern Pacific region and the Indo-West Pacific Region, and is the only bucephalid genus which has “apparently strongly radiated within the Epinephelinae”. Since that paper [8] our knowledge of epinepheline bucephalids has increased markedly [2, 3, 5] reinforcing that point, but suggesting that *Neidhartia* has also radiated, at least in the Indo-West Pacific region. The morphological distinctions between *Prosorhynchus* and *Neidhartia* are minor, but molecular evidence [3] indicates that these distinctions are reflected by the molecules. Those species of *Prosorhynchus* with a variable ovary configuration (e.g., *P. epinepheli*, *P. longisaccatus*) may invalidate this distinction, or may belong to either monophyletic genus.
